# Potential of Ko-Klan Traditional Thai Remedy for Evaluation of Antioxidant and RT-PCR Anti-Inflammatory Activities

**DOI:** 10.1155/sci5/4361994

**Published:** 2025-11-24

**Authors:** Sutthichat Kerdphon, Pariya Atawong, Sukanya Reanpang, Phanupong Changtor, Nopawit Khamto, Gorawit Yusakul, Nitra Nuengchamnong, Kittisak Buddhachat, Jira Jongcharoenkamol

**Affiliations:** ^1^Department of Chemistry, Faculty of Science, Naresuan University, Phitsanulok 65000, Thailand; ^2^Center of Excellence for Innovation in Chemistry (PERCH-CIC), Naresuan University, Phitsanulok 65000, Thailand; ^3^Center of Excellence for Innovation and Technology for Detection and Advanced Materials, Naresuan University, Phitsanulok 65000, Thailand; ^4^Center of Excellence for Natural Health Product Innovation, Naresuan University, Phitsanulok 65000, Thailand; ^5^Department of Pharmaceutical Chemistry and Pharmacognosy, Faculty of Pharmaceutical Science, Naresuan University, Phitsanulok 65000, Thailand; ^6^Department of Biology, Faculty of Science, Naresuan University, Phitsanulok 65000, Thailand; ^7^Department of Biochemistry, Faculty of Medical Science, Naresuan University, Phitsanulok 65000, Thailand; ^8^Research and Innovation Cluster for Natural Health Products, Naresuan University, Phitsanulok 65000, Thailand; ^9^Science Lab Centre, Faculty of Science, Naresuan University, Phitsanulok 65000, Thailand

**Keywords:** anti-inflammation, antioxidant, gene expression, herbal medicine, lipoxygenase, *Mallotus repandus*

## Abstract

*Ko-klan* is a traditional Thai herbal medicine that is officially recognized for treating muscle pain; however, its mechanism of action has not been characterized. This study investigated the antioxidant and anti-inflammatory properties of three Ko-klan remedy formulations and identified potential bioactive markers using molecular docking. Ko-klan formulations were extracted using decoction, maceration, ultrasound-assisted extraction (UAE), and microwave-assisted extraction (MAE), with ethanol as the solvent. Phytochemical analyses were performed to determine the total phenolic content (TPC) and total flavonoid content (TFC), with antioxidant activity evaluated using the DPPH, ABTS, and FRAP assays. The anti-inflammatory effects were assessed by gene expression analysis (TNF-*α*, iNOS, COX-2, and IL-1*β*) and inhibition of nitric oxide production, and lipoxygenase (LOX) activity. LC-ESI-QTOF-MS/MS identified 175 compounds, with molecular docking performed against 5-LOX. Formulation-3 exhibited notable bioactivity, with a TPC of up to 1.08 g GAE/g and a TFC of 7.30 mg QE/g, and IC_50_ values of 51.8 μg/mL for DPPH and 92.3 μg/mL for ABTS assays, 7 to 10 fold than the Trolox standard. Anti-inflammatory activity showed comparable inhibition of nitric oxide production to the L-NAME standard and effective LOX inhibition at 62.7 μg/mL. The MAE-based Ko-klan remedy extract significantly suppressed LPS-induced inflammatory gene expression, comparable to dexamethasone. Molecular docking showed that caffeoyl quinic acid and brazilin were potent 5-LOX inhibitors with binding energies of −10.14 and −10.24 kcal/mol, respectively. Results demonstrate that Ko-klan remedies, particularly formulation-3, possessed significant antioxidant and anti-inflammatory activities, with phytochemical richness, effective suppression of inflammatory mediators, and potential bioactive markers such as caffeoyl quinic acid and brazilin, thereby supporting their traditional use and providing a scientific basis for further therapeutic development.

## 1. Introduction

*Mallotus repandus* (Rottler) Müll.Arg, a key herbal component in the three Ko-klan remedy formulations, is recognized for its traditional use in relieving muscle pain, and was officially listed as part of the Ko-klan remedy in the Thai National List of Essential Medicine in 2021 [1]. However, the mechanisms of action of this herb remain underexplored. Evaluating the bioactivity and chemical constituents is crucial for the development and quality control of medicines based on Ko-klan remedy formulations.

The Ko-klan remedy comprises three formulations, each containing different types of herbs and varying herbal ratios (Table 1) [1]. This remedy is traditionally prescribed for alleviating musculoskeletal pain. According to the Thai National List of Essential Medicines, the formulation can be administered in two dosage forms: (i) as an infusion, in which 1 g of the powdered preparation is steeped in 120–200 mL of hot water and consumed three times daily before meals, and (ii) as a decoction, in which the crude materials are boiled with water until the volume is reduced to one-third, with 120–200 mL of the extract administered three times daily before meals. The herbs used in the Ko-klan remedy and their chemical constituents have been reported to exhibit anti-inflammatory and antioxidant bioactivities. Bergenin and mallorepine found in *M. repandus* demonstrated strong antioxidant and anti-inflammatory properties [2, 3], while terpenoids from *Elephantopus scaber* L., rhinacanthins from *Rhinacanthus nasutus* (L.) Kurz, and compounds in *Caesalpinia sappan* L. extracts, including brazilin, flavonoids, and protosappanins inhibited nitric oxide (NO) synthesis and reduced levels of inflammatory cytokines [3–5]. Volatile oils and alkaloids present in *Aegle marmelos* (L.) Corrêa and *Piper interruptum* Opiz also exhibited antioxidants, anti-inflammatory, and analgesic activities [6–8]. The extraction method and choice of solvent play critical roles in isolating bioactive compounds from herbal materials and enhancing the quality of the crude extract. Optimized crude extracts can be developed into various dosage forms that offer improved patient compliance and promote the integration of traditional Thai medicine into modern healthcare.

Despite extensive traditional use and studies on the chemical constituents and bioactivities of individual constituent herbs, the Ko-klan remedy lacks comprehensive scientific validation in modern medicine. Current research gaps include a limited understanding of the underlying mechanisms of action and chemical profile, leading to insufficient quality control standards for Ko-klan remedy extracts. To address these limitations, this study investigated the antioxidant and anti-inflammatory properties of three traditional Ko-klan remedies, elucidated their mechanisms of action, and analyzed their chemical profiles to identify potential bioactive markers for future standardization of these remedies. Three formulations of the Ko-klan remedy were evaluated for their chemical constituents and bioactivities. Various extraction methods, including decoction, hot ethanol extraction, maceration, ultrasound-assisted extraction (UAE), microwave-assisted extraction (MAE), and solvent types, were compared for their impact on phytochemical content and biological activity. All the extracts were analyzed for total phenolic content (TPC) and total flavonoid content (TFC), with antioxidant activity assessed by the DPPH, ABTS, and FRAP assays. Anti-inflammatory effects were examined by gene expression analysis, NO production, and lipoxygenase (LOX) inhibition. The findings support the use of the Ko-klan remedy for muscle pain relief and provide a foundation for its standardization and quality control.

## 2. Materials and Methods

### 2.1. Plant Material and Preparation of the Ko-Klan Remedy

Dried plant materials of *Mallotus repandus* (Rottler) Müller. Arg. (stem), *Elephantopus scaber* L. (whole plant), *Aegle marmelos* (L.) Corrêa (fruit), *Rhinacanthus nasutus* (L.) Kurz (whole plant), *Cryptolepis buchananii* Roem. and Schult. (stem), *Piper interruptum* Opiz (fruit), and *Caesalpinia sappan* L. (wood) were certified and provided by Charoensuk Pharma Supply Co., Ltd., Thailand. The herbal materials were then ground into a fine powder. Three formulations of the Ko-klan remedy were prepared following the proportions described in the Thai National List of Essential Medicines (2021) (Table 1) [1].

### 2.2. Extraction of the Ko-Klan Remedies

Traditional Ko-klan remedies are typically prepared by either infusion or decoction. In this study, extracts of Ko-klan remedy formulations were prepared using decoction, maceration, UAE, and MAE methods (Table 2). Each formulation was boiled at a solid-to-solvent ratio of 1:10 for 30 min. Extraction was conducted using the same procedure; however, ethanol was employed instead of water, a method referred to as hot ethanol extraction. For the maceration process, each Ko-klan remedy was immersed in a solid-to-solvent ratio of 1:10 in a sealed container and maintained at room temperature for 24 h. The extracts were then filtered through filter paper and evaporated. In the case of UAE, an ultrasonic cleaner, Elmasonic model Select 180, with a frequency of 37 kHz was used. Each Ko-klan remedy was immersed in the solvent at a solid-to-solvent ratio of 1:10 and subjected to sonication at a temperature range of 30°C–40°C for 30 min. The resultant extracts were filtered through a filter paper and evaporated. For MAE, Microwave Green Extraction of Natural Products, Milestone ETHOS X was followed in a PTFE vessel. Each Ko-klan remedy was placed in the solvent at a solid-to-solvent ratio of 1:10 and extracted using at microwave power of 500 W for 30 min. The obtained extracts were filtered through filter paper and evaporated. The percentage yield was determined using the following equation: yield (%) = (weight of extract/weight of dried Ko-klan remedy powder used) × 100.

### 2.3. Antioxidant Activity Assays

#### 2.3.1. DPPH Radical Scavenging Assay

The protocol was modified from the method described by Suphrom and Insumrong [9]. Sample solutions of the Ko-klan remedy crude extracts (10–4000 μg/mL) were prepared in an extraction solvent or Trolox (10–1000 μg/mL, positive control) of 50 μL mixed with 150 μL of 0.3 mM DPPH in 95% ethanol in a 96-well plate. The mixture was incubated in the dark for 30 min before measuring the absorbance at 517 nm. Antioxidant activity was calculated using the following formula:

(1) $$\begin{array}{c} \%\text{DPPH scavenging activity}=\left[\frac{\left[{\text{Abs}}_{\text{control}}-\left({\text{Abs}}_{\text{sample}}-{\text{Abs}}_{\text{blank}}\right)\right]}{{\text{Abs}}_{\text{control}}}\right]\times 100, \end{array}$$

where Abs_control_ = absorbance of the control, Abs_sample_ = absorbance of the Ko-klan remedy extracts or Trolox, and Abs_blank_ = absorbance of the sample blank.

The half-maximal inhibitory concentration (IC_50_) of each Ko-klan remedy and Trolox was determined by plotting the percentage of radical scavenging activity versus the logarithm (log) of the final concentration of the sample (μg/mL).

#### 2.3.2. ABTS Radical Scavenging Assay

The ABTS radical-scavenging activity was modified from the method reported by Re and Pellegrini [10]. Briefly, the ABTS radical cation (ABTS^.+^) was diluted with 95% ethanol to give an absorbance of 0.70 (±0.02) at 734 nm. Next, 20 μL of each Ko-klan crude extract (100–10,000 μg/mL) or Trolox (10–1000 μg/mL) was added to 180 μL of ABTS^.+^ working solution. The mixture was incubated in the dark at room temperature for 6 min before measuring the absorbance of the reaction mixture at 734 nm using a microplate reader. The antioxidant activity was calculated using the following formula:

(2) $$\begin{array}{c} \%\;{\text{ABTS}}^{.+}\text{ scavenging activity}=\left[\frac{\left[{\text{Abs}}_{\text{control}}\;–\;\left({\text{Abs}}_{\text{sample}}\;–\;{\text{Abs}}_{\text{blank}}\right)\right]}{{\text{Abs}}_{\text{control}}}\right]\times 100, \end{array}$$

where Abs_control_ = absorbance of the control, Abs_sample_ = absorbance of the Ko-klan remedy extracts or Trolox, and Abs_blank_ = absorbance of the sample blank.

The half-maximal IC_50_ values of each Ko-klan remedy and Trolox were determined by plotting the percentage of radical scavenging activity against the logarithm (log) of the final concentration of the sample (μg/mL).

#### 2.3.3. FRAP Assay

The FRAP assay was modified from the method described by Müller and Fröhlich [11]. To prepare the FRAP reagent, 300 mM acetate buffer (pH 3.6), 10 mM 2,4,6-tripyridyl-s-triazine (TPTZ) in 40 mM HCl, and 20 mM FeCl_3_•6H_2_O were mixed at a ratio of 10:1:1 (by volume). The reaction was performed by combining 20 μL of each Ko-klan remedy crude extract (0.01–1000 μg/mL) or Trolox with 180 μL of a freshly prepared FRAP working solution. The reaction mixture was incubated in the dark at room temperature for 30 min before measuring the absorbance of the mixture at 593 nm. Standard FeSO_4_•7H_2_O solutions were used to construct the calibration curves. The calibration curve was represented by a linear regression equation: *y* = 1.2128*x*, *R*^2^ = 0.9993. The antioxidant activity was reported as milligrams of Fe^2+^ per gram of crude extract (mg Fe^2+^/g Et).

### 2.4. TPC

The Folin–Ciocalteu assay was used to determine the TPC of the Ko-klan remedy extracts, following the protocol described by Elin Novia and Berna [12] with some modifications. Briefly, 25 μL of the crude working Ko-klan remedy extract solution (2 mg/mL) was combined with 225 μL of 10%v/v Folin–Ciocalteu reagent. The mixture was incubated for 5 min, followed by the addition of 25 μL of 1 M Na_2_CO_3_ solution. The mixture was then incubated in the dark for 60 min, and the absorbance of the solution was determined at 756 nm using a microplate reader. Standard gallic acid (GA) solutions were used for the calibration curve. The calibration curve was represented by a linear regression equation: *y* = 0.0076*x* − 0.0286, *R*^2^ = 0.9978. The TPC was reported as grams of GA equivalent per gram of crude extract (g GAE/gEt).

### 2.5. TFC

The TFC was determined following the procedure described by Ngamdokmai and Ingkaninan [5], with some modifications. Briefly, 50 μL of crude Ko-klan remedy extract solution (4 mg/mL) was combined with 150 μL of 95% ethanol, 10 μL of 1 M CH_3_COONa, and 10 μL of 10% AlCl_3_. The reaction mixture was incubated in the dark at room temperature for 40 min. The absorbance was measured at 415 nm using a microplate reader. Standard quercetin solutions were used to construct the calibration curve, which was represented by a linear regression equation: *y* = 0.0227*x* − 0.0169, *R*^2^ = 0.9992. The TFC was reported as milligrams of quercetin equivalent (QE) per gram of crude extract (mg QE/g Et).

### 2.6. Cell Viability

Cell viability and anti-inflammation were performed as described previously [13, 14]. The biological activities on RAW 264 cells (from RIKEN BioResource Research Center, Japan) were used to determine cell viability and anti-inflammation, with biosafety approval granted under NUIBC OT 66-11-45 (approval number 67-01) from the Naresuan University Institutional Biosafety Committee (NUIBC).

The RAW 264 cells were cultured in Dulbecco's Modified Eagle Medium (DMEM) with 100 U/mL penicillin, 100 μg/mL streptomycin (1% pen-strep), 0.1% amphotericin B, and 10% fetal bovine serum (FBS) and incubated at 37°C under 5% CO_2_. A total of 30,000 cells were seeded in a 96-well plate and incubated at 37°C in 5% CO_2_ for 24 h. The medium was removed and replaced with fresh medium and different concentrations of Ko-klan crude extracts. The final DMSO concentration for treating cells was 0.4%, and the concentrations of the Ko-klan remedy extracted were between 1.95 and 31.25 μg/mL. All the cells were incubated for 20 h, followed by the addition of 3-(4,5-dimethylthiazol-2-yl)-2,5-diphenyltetrazolium bromide (MTT) solution to a 0.5 mg/mL final concentration. After 4 h, the mixture was removed, and 150 μL of DMSO: ethanol (1:1) was added to dissolve the formazan. The 96-well plate was shaken on a microplate shaker at 400 rpm for 5 min. The absorbances of the mixtures were measured by a microplate reader at 570 nm. Cell viability was calculated using the following formula:

(3) $$\begin{array}{c} \%\text{ viability}=\left[\frac{{\text{Abs}}_{\text{sample}}}{{\text{Abs}}_{\text{control}}}\right]\times 100, \end{array}$$

where Abs_control_ = absorbance of the control and Abs_sample_ = absorbance of the Ko-klan extract.

### 2.7. Gene Expression Measurement

RAW 264 cells were seeded in a 24-well plate with DMEM, 1% penicillin-streptomycin, 1% amphotericin B, and 10% FBS at 4 × 10^5^ cells per well. The cells were incubated at 37°C in CO_2_ for 24 h, and the medium was removed. Next, 15.6 μg/mL Ko-klan remedy in DMEM with 1% pen-strep and 0.1% amphotericin B were added and incubated at 3°C–7°C in CO_2_ for 2 h, followed by adding LPS (100 ng/mL) and incubating at 37°C in CO_2_ for 16 h. The mixture was removed and washed twice with PBS (1 mL). mRNA was extracted using the RNAspin Mini RNA Isolation Kit with 600 μL of lysis buffer and 6 μL of *β*-mercaptoethanol. The mRNA was then converted into cDNA in vitro using an iScript cDNA Synthesis Kit.

Gene expressions of the GAPDH housekeeping gene, TNF-*α*, COX-2, iNOS, and IL1-*β* as shown in Table S3 were assessed by quantitative real-time PCR (qRT-PCR). The reaction mixture was prepared with 1X SensiFAST SYBR No-ROX Kit, 0.2 μM of each primer, 1 μL of 1:3 cDNA, and nuclease-free water to a final volume of 20 μL. Amplification was performed on a real-time PCR machine (Bioer, China) with an initial denaturation at 95°C for 5 min, followed by 40 cycles at 95°C for 30 s and 60°C for 30 s, and a final extension at 72°C for 7 min. Fluorescence data were collected at the end of each 60°C step during the PCR cycles. All samples were subjected to melting curve analysis to determine the specificity of the PCR amplification. The primers and qRT-PCR conditions for the TNF-*α*, COX-2, iNOS, IL1-*β*, and GAPDH genes are detailed in Table S3. The PCR products were detected using a UV transilluminator (Vileber Lourmat, France). The cycle threshold (Ct) was measured. GAPDH was used as an internal reference for normalization, and then calculated as the relative expression using the following formula:

(4) $$\begin{array}{c} \text{Gene expression calculation }2^{-\Delta \Delta \text{Ct}},\\ \Delta C_{t\text{ sample}}=C_{t\text{ target}}^{s}-C_{t\text{ housekeeping}}^{s},\\ \Delta C_{t\text{ LPS}}=C_{t\text{ target}}^{L}-C_{t\text{ housekeeping}}^{L},\\ \Delta \Delta C_{t}=\Delta C_{t\text{ sample}}-\Delta C_{t\text{ LPS}},\\ \text{Fold difference}=2^{-\Delta \Delta \text{Ct}}. \end{array}$$

### 2.8. NO Colorimetric Assay

The RAW 264 cells were seeded into a 96-well plate with DMEM, 1% penicillin-streptomycin, 0.1% amphotericin B, and 10% FBS at a density of 1.5 × 10^5^ cells per well. The cells were incubated at 37°C in a CO_2_ atmosphere for 24 h, after which the medium was removed. Different concentrations of Ko-klan remedy extracts in fresh medium were added, followed by a 2 h incubation. Subsequently, 1 μg/mL LPS was added, and the cells were incubated for another 24 h. After incubation, 50 μL of medium was transferred to a new 96-well plate. A 1% sulfanilamide solution in 5% phosphoric acid was added, and the mixture was incubated in the dark for 10 min. Next, 0.1% N-(1-naphthyl) ethylenediamine (NED) was added, and the mixture was incubated for another 10 min in the dark. The absorbance of the solution was measured at 550 nm using a microplate reader. A standard curve was prepared using 0–100 μM sodium nitrite solution instead of the medium.

### 2.9. Soybean LOX Inhibition Assay

The soybean LOX inhibitory activity was examined following the procedure described by Pontiki and Hadjipavlou-Litina in 2007 [15] with some modifications. The Ko-klan crude extracts were dissolved in the extraction solvent at different concentrations. Then, 10 μL of each sample was mixed with 20 μL of 6 mM sodium linoleate and 170 μL of soybean LOX solution in 0.1 M phosphate buffer (pH 8.0) (enzyme activity 80–120 *V*_mean_) at cold temperature. The conversion of sodium linoleate to 13-hydroperoxy linoleic acid was measured at 234 nm (*V*_mean_). Nordihydroguaiaretic acid (NDGA) and ethanol were used as positive and negative controls, respectively. LOX inhibition activity was calculated using the following formula:

(5) $$\begin{array}{c} \%\text{Lipoxygenase inhibition}=\left[\frac{\left(V_{\text{mean}}\text{control }–V_{\text{mean}}\text{sample}\right)}{V_{\text{mean}}\text{control}}\;\right]\times 100, \end{array}$$

where *V*_mean_control is the mean velocity of the control, and *V*_mean_sample is the mean velocity of the Ko-klan remedy extracts or NDGA.

### 2.10. Statistical Analysis

All the experimental data were expressed as mean values with corresponding standard deviations (SD), derived from a minimum of three independent experiments. Statistical analyses were performed using one-way analysis of variance (ANOVA), followed by Tukey's post hoc test for multiple comparisons. Analyses were conducted using IBM SPSS Statistics for Windows, Version 21.0 (IBM Corp., Armonk, NY, USA). A *p* value of ≤ 0.05 was considered statistically significant. This threshold reflected the conventional 95% confidence level commonly applied in biomedical research to balance the rigor and the interpretability of experimental outcomes [16].

### 2.11. Liquid Chromatography-Electrospray Ionization-Quadrupole Time-of-Flight Mass Spectrometry (LC-ESI-QTOF-MS/MS) Analysis

The chemical constituents in the Ko-klan remedy extracts were analyzed using LC-ESI-QTOF-MS/MS on an Agilent 6540 UHD Accurate Mass-QTOF-LC/MS system (Agilent Technologies, Singapore). The ethanolic extract (10 μL) was separated using reversed-phase high-performance liquid chromatography (HPLC 1260 series) with a Luna C-18 column (4.6 × 150 mm, 5 μm; Phenomenex, USA). The mobile phase included 0.1% formic acid in water (A) and 0.1% formic acid in acetonitrile (B), with a gradient elution from 95:5 to 5:95 (A:B, v/v) over 30 min, maintained for another 10 min. The column was kept at 35°C with a flow rate of 0.5 mL/min. Mass spectra were recorded in both positive and negative ESI modes across a range of m/z 100–1000 Da, with a scan rate of 250 ms/spectrum. Instrument settings included a nitrogen flow rate of 10 L/min, gas temperature of 350°C, nebulizer pressure of 30 psig, capillary voltage of 3500 V, fragmentor voltage of 100 V, skimmer voltage of 65 V, and octopole RFP of 750 V. Untargeted MS/MS data were acquired using three collision energies of 10, 20, and 40 V. Data processing was performed using MassHunter software (Agilent Technologies, CA, USA), with peak identification based on retention times, mass spectra, and fragmentation patterns compared to the published literature, the MassHunter Metlin Metabolite PCD/PCDL, and the Human Metabolome Database (HMDB) (https://www.hmdb.ca).

### 2.12. Molecular Docking

#### 2.12.1. Protein and Ligand Preparations

The structure of arachidonate 5-LOX (5-LOX) in a complex with NDGA was retrieved from the RCSB Protein Databank (PDB ID: 6N2W) [17]. The structure was cleaned by removing the water molecules and ions using BIOVIA Discovery Studio 2021 Visualizer (Dassault Systèmes, San Diego). The protein chain B was selected for this study. Following the full-length sequence (UniProt P09917), the missing residues were modeled using AlphaFold3 including residues 1–22, 59–61, 188–232, 312–321, and 434–447 [18]. The modeled missing residues were linked to the crystal structure via amide bonds, followed by minimizing the energy using the AMBER10:EHT forcefield within the Molecular Operating Environment (MOE) 2015 software. The completed structure was exported in PDB format. 3D structures of the ligands were generated using Gaussview6 with protonation state correction at pH 8.0, using MarvinSketch software. The structures were fully optimized by the DFT calculation at B3LYP/6-31G (d,p) level of theory using the Gaussian16 package [19] and saved in PDB format.

#### 2.12.2. Molecular Docking

Molecular docking was performed using GNINA with deep learning version 1.3 [20], running on Google Colab. The grid box was automatically generated using the Cartesian coordinates of the co-crystallized inhibitor, NDGA, with an additional spacing of 8 Å in all dimensions. The exhaustiveness was set to 32 for all the predictions, with CNN affinity used to select the binding poses for the visualization analysis.

## 3. Results and Discussion

### 3.1. Extraction Yields and Phytochemical Analysis

Formulation-1 of the Ko-klan remedy powder was extracted using deionized water (T11, decoction) and ethanol (T12, hot ethanol), yielding 11.2% and 4.89%, respectively (Table 2), with differences also observed in the extraction yield, TPC, TFC, and antioxidant activities measured by the ABTS, DPPH, and FRAP assays. Extract T12 exhibited higher TFC at 4.30 ± 0.35 mg QE/g and higher antioxidant activity as measured by the FRAP assay at (0.685 ± 0.085) × 10^2^ mg Fe^2+^/g, compared to T11. Therefore, ethanol was selected as the optimal solvent for extracting Ko-klan. Various extraction methods (maceration, UAE, and MAE) were applied using ethanol, and the three formulations of Ko-klan were compared (Table 3). Formulation-1 extracts T12–T15 showed yields ranging from 1.95% to 7.47%, with formulation-2 extracts T22 to T25 yielding between 2.78% and 4.58%, whereas formulation-3 (T32 to T35) produced yields from 1.27% to 3.89%. These results suggested that MAE may enhance molecular diffusion and facilitate compound release from herbal matrices [21], thereby improving the extraction yield, especially in formulations-1 and -3.

The most abundant secondary metabolites in plants usually contain phenolic and flavonoid compounds that act as antioxidants by chelating ions, scavenging free radicals, and exerting anti-inflammatory activities [22, 23]. The ethanolic extracts of Ko-klan remedy formulation-3 exhibited high TPC, with values reaching 1.08 g GAE/g extract (Table 3, T32 and T35). This elevated TPC was consistent with the presence of phenolic-rich constituents derived from *C. sappan*, *C. buchananii*, and *P. interruptum*, which are unique to this formulation and known for their abundant phenolic content [24–26]. On the other hand, formulation-1 and -2 of the Ko-klan remedy, which did not contain *C. sappan*, *C. buchananii*, and *P. interruptum*, recorded TPC values ranging from 0.349 to 0.597 g GAE/g (Table 3, T12–T25). Diverse extraction methods yielded significantly different TPC for the same formulation of the Ko-klan remedy, particularly MAE. The TFC in ethanolic Ko-klan remedy extracts ranged from 1.62 to 7.30 mg QE/g (Table 3). In this study, MAE improved the trend of total flavonoids in formulations-2 and -3 of the Ko-klan remedy.

### 3.2. Antioxidant Activity

#### 3.2.1. DPPH Assay

The antioxidant capacity was expressed as the IC_50_ value, which indicates the concentration required to inhibit 50% of DPPH free radical activity. The IC_50_ value of the Trolox standard—used as a positive control—was 0.104 ± 0.001 × 10^2^ μg/mL (Table 3). Formulation-1 of the Ko-klan remedy extract exhibited DPPH antioxidant activities ranging from 0.942 ± 0.064 × 10^2^ to 2.38 ± 0.07 × 10^2^ μg/mL. In this case, MAE increased the DPPH antioxidant activity twofold compared to the other methods (Table 3, T12 – T15). In formulation-2, all the ethanolic extracts had similar antioxidant activity to the first formulation, ranging from 0.912 ± 0.025 × 10^2^ to 1.95 ± 0.02 × 10^2^ μg/mL (Table 3, T22–T25). Interestingly, extracts from formulation-3 of the Ko-klan remedy containing high TPC showed DPPH antioxidant activity with IC_50_ < 80 μg/mL (Table 3, T32 – T35); in particular, T34 had an IC_50_ of 51.8 μg/mL while the other formulation showed lower TPC and antioxidant activity. Therefore, TPC can be used as an indicator of antioxidant activity.

#### 3.2.2. ABTS Assay

The ABTS antioxidant assay relies on the ability of antioxidants to decrease the dark green color of ABST radicals. Formulations-1 and -2 of the Ko-klan remedy extracts inhibited the ABTS radical activity, with IC_50_ values ranging from 1.17 ± 0.15 × 10^2^–2.46 ± 0.01 × 10^2^ μg/mL (Table 2, T12–T25) while formulation-3 showed higher ABTS antioxidant activity with an IC_50_ < 100 μg/mL (Table 3, T32–T35). These experiments used Trolox as a positive control, which displayed IC_50_ of inhibitory ABTS activity at 0.089 ± 0.001 × 10^2^ μg/mL.

#### 3.2.3. FRAP Assay

The antioxidant efficacy was measured using the FRAP assay redox reaction. The antioxidants in the extracts acted as reducing agents at low pH and reduced the ferric tripyridyl triazine (Fe^3+^-TPTZ) complex to blue-violet ferrous form (Fe^2+^-TPTZ). The FRAP assay revealed that formulation-3 of the Ko-klan remedy exhibited the highest antioxidant activity compared to the other formulations, ranging from 1.23 ± 0.06 × 10^2^ to 1.65 ± 0.01 × 10^2^ mg/mL mgFe^2+^/g, while the Trolox standard gave 0.765 ± 0.150 × 10^2^ mgFe^2+^/g. This result suggested that the antioxidant activity of the Ko-klan remedy extracts did not pass through the redox reaction.

The DPPH and ABTS results indicated that the antioxidant activity correlated with TPC. Therefore, the TPC assay can be used as an initial quality control of antioxidant extracts.

### 3.3. Cell Viability

**Figure 1:**
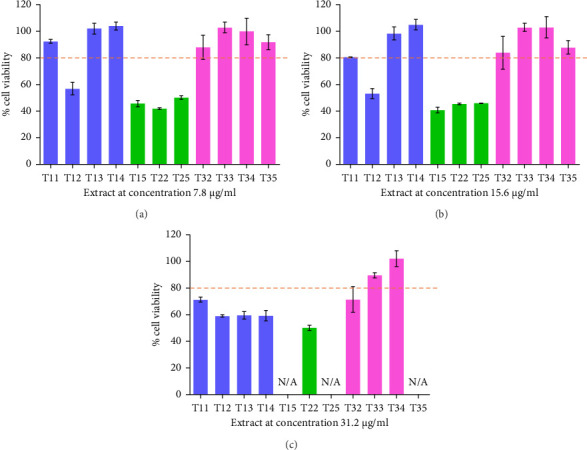
Cell viability (%) of Ko-klan remedy extracts on RAW 264 cells at concentrations of 7.8 μg/mL (a), 15.6 μg/mL (b), and 31.2 μg/mL (c). N/A; the extracts were not tested for cell viability due to the lowest concentration of less than 40%.

The cell viability of each extract was assessed using the MTT assay to determine the highest concentration that maintained viability above 80% in RAW 264 cells. This concentration was then used to evaluate the anti-inflammatory properties of the Ko-klan medicinal remedy (Figure 1 and Table S1). At 31.2 μg/mL, only two extracts T33 and T34 exhibited cell viability above 80% compared to the control. Therefore, a lower concentration of 15.6 μg/mL was further tested. Results revealed that extracts T11, T13, T14, T32, T33, T34, and T35 maintained greater than 80% cell viability. By contrast, all the other extracts showed cytotoxicity even at concentrations below 1.96 μg/mL. Extracts from formulation-2, containing a higher proportion of *M. repandus*, demonstrated cytotoxic effects, suggesting a correlation between cell viability and the *M. repandus* proportion in the extracts. Based on these findings, seven extracts from formulations T11, T13, T14, T32, T33, T34, and T35 at 15.6 μg/mL were selected for further investigation of their anti-inflammatory activities.

### 3.4. Effect of Ko-Klan Remedy Extracts on iNOS, TNF-α, COX-2, and IL-1β mRNA Expressions

**Figure 2:**
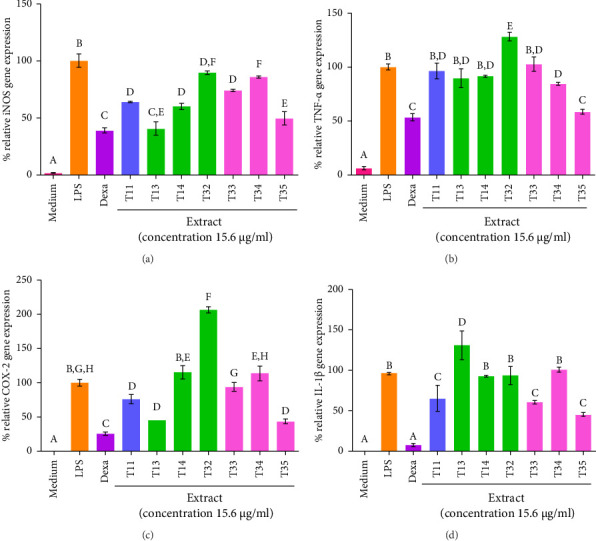
Effect of Ko-klan remedy extracts on (a) iNOS, (b) TNF-α, (c) COX-2, and (d) IL-1β mRNA expressions. Cells were treated with lipopolysaccharide (LPS, 100 ng/mL) and ethanolic Ko-klan extracts (15.6 μg/mL) or a blank. Dexamethasone (dexa) was treated at 62.5 μM. Different letters above the bars indicate statistically significant differences between the treatment groups, while the same letters indicate no significant difference in extraction yield. Statistical significance was determined using one-way ANOVA followed by Tukey's post hoc test (*p* < 0.05).

The cell viability results showed that the concentration of 15.6 μg/mL of ethanolic Ko-klan remedy extracts from formulations-1 and -3 (T11, T13, T14, T32, T33, T34, and T35) exhibited high cell viability at > 80%, and these were used for investigating the gene expression level of inflammatory-responsive genes including iNOS, TNF-*α*, COX-2, and IL-1*β* in LPS-stimulated RAW 264 cells. The relative gene expression levels of iNOS, TNF-*α*, COX-2, and IL-1*β* were assessed, using the LPS-treated group as a reference (defined as 100% relative gene expression) (Figure 2).

For iNOS gene expression, extracts T11, T13, and T14 showed the ability to reduce the LPS-stimulated iNOS gene expression levels, ranging from 40.8% to 64.2% compared to only the LPS treatment. Treatment with formulation-3 extracts displayed weaker downregulation of the iNOS gene expression compared to formulation-1, except for the T35 extract, which showed a strong 49.6% downregulation of iNOS gene expression, representing 50.4% inhibition compared to the LPS control. Among the extraction methods, maceration (T13) demonstrated the optimal inhibitory effect, reducing relative iNOS mRNA expression to 40.8%, similar to that observed with dexamethasone at 39.0% (Figure 2(a), Tables S5 and S11).

Most of the Ko-klan remedy extracts (T11, T13, T14, and T32-34) did not decrease the gene expression of TNF-*α*; however, the T35 extract (MAE) showed a strong 58.6% reduction of TNF-*α* expression, comparable to dexamethasone (Figure 2(b), Tables S4 and S12).

COX-2 plays a vital role in regulating the production of the inducible COX-2 enzyme to synthesize prostaglandin leading to possible inflammation in various tissues [27]. Treatment with the T11 extract, prepared by decoction in deionized water, resulted in a 76.4% reduction of COX-2 gene expression relative to the LPS-treated control group. The ethanol maceration method effectively enhanced the inhibition of COX-2 mRNA expression, resulting in a relative expression of 45.2% (T13). By contrast, UAE showed no inhibition of COX-2 gene expression (T14). Interestingly, formulation-3 extracted using MAE showed increasing inhibition of COX-2 gene formation, with 43.9% of the relative COX-2 mRNA expression (T35), while the other methods did not affect the COX-2 mRNA expression (T32-34) (Figure 2(c), Table S7 and S13). Therefore, the maceration extract of formulation-1 and MAE of formulation-3 were potential candidates as anti-inflammatory agents for pharmaceutical or cosmeceutical products.

The inhibition of the IL-1*β* gene expression was also studied (Figure 2(d), Table S6 and S14). Formulation-1 of the Ko-klan remedy (T11) showed relative IL-1*β* gene expression at 65.29%. The ethanolic extracts of this formulation were also investigated, with no inhibition of this gene expression observed in T13 and T14. Formulation-3 of the Ko-klan remedy using maceration and MAE extracts (T33 and T35) showed decreased IL-1*β* mRNA gene expression of 60.72% and 45.29% compared to LPS, while heating (decoction) and UAE did not impact the IL-1*β* gene expression.

Our findings demonstrated that the Ko-klan remedy extracts, especially T35, showed promise for anti-inflammation, as evidenced by the downregulation of inflammation-related genes including iNOS, TNF-*α*, COX-2, and IL-1*β* with comparable ability to dexamethasone. IL-1*β* and TNF-*α* are potent pro-inflammatory cytokines that stimulate signaling pathways such as NF-κB and MAPKs, leading to the transcriptional activation of iNOS and COX-2 [28]. Upregulation of iNOS results in excessive NO production [29], while COX-2 enhances prostaglandin synthesis [27] and both sustain and amplify inflammatory responses. Thus, the strong link between IL-1β/TNF-α and iNOS/COX-2 expression reflects their central role in driving chronic inflammation.

Ko-klan remedy extracts have not been previously investigated for anti-inflammatory activity but individual herbal components such as *C. buchananii*, *C. sappan*, and *Piper interruptum* have been reported to contain compounds that inhibit inflammatory gene expression [5, 7, 30]. The anti-inflammatory potential of *C. buchananii* 50% ethanolic extract inhibited cyclooxygenase and 5-LOX in rat leukocytes, suppressed TNF-α production by 52.19% in human monocytic cells comparable to dexamethasone, and showed anti-edematous activity in carrageenan-induced rat paw edema [31]. *C. buchananii* extract showed significant analgesic, anti-inflammatory, and chondroprotective activities [5]. The extract reduced acetic acid-induced writhing by 31.25%–50.53% at 60–250 mg/kg [5]. *C. buchananii* extract inhibited EPP-induced ear edema and carrageenan-induced paw edema dose-dependently, and demonstrated chondroprotective effects by reducing glycosaminoglycan and hyaluronan release while preserving cartilage matrix and suppressing MMP-2 activity [5]. Five compounds from *C. sappan* heartwood showed anti-inflammatory activities in macrophages and chondrocytes. All fractions inhibited proinflammatory cytokines IL-6 and TNF-α [32]. Brazilin was the most potent, with IC_50_ values of 18 μM (IL-6) and 29 μM (TNF-α) in macrophages, and 5 and 3 μM in chondrocytes [32], while sappanol increased anti-inflammatory IL-10 secretion [32]. All these compounds showed minimal cytotoxicity [32].

The anti-inflammatory properties observed in formulation-3 of the Ko-klan remedy are well-supported by the documented bioactive compounds in its individual herbal constituents. These findings provided strong evidence that the regulation of inflammatory gene expression constituted the primary mechanism underlying the therapeutic effects of formulation-3. When formulation-3 was processed using MAE, the resulting extract exhibited enhanced anti-inflammatory bioactivity compared to the other extraction methods, demonstrating the critical role of extraction methodology in optimizing compound bioavailability. MAE represented a superior extraction approach for producing anti-inflammatory Ko-klan remedy extracts, offering significant potential for pharmaceutical development and clinical applications.

### 3.5. Inhibition of NO Production of Ko-Klan Remedy Extracts

**Figure 3:**
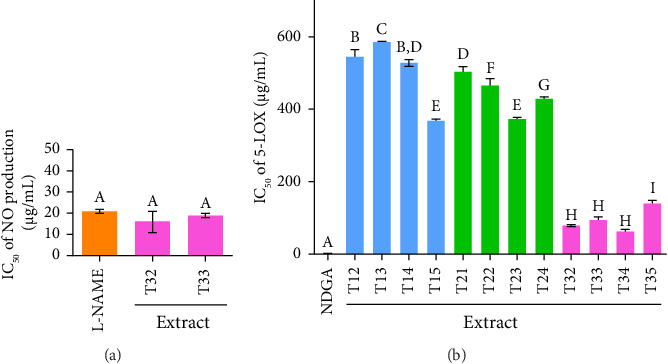
Anti-inflammatory activity of Ko-klan remedy extracts: (a) nitric oxide (NO) inhibitory assay; (b) soybean lipoxygenase (LOX) inhibition assay. Different letters above the bars indicate statistically significant differences between treatment groups, while the same letters indicate no significant difference in extraction yield. Statistical significance was determined using one-way ANOVA followed by the least significant difference (LSD) post hoc test (*p* < 0.05).

The NO production was assayed to test the iNOS gene activity of Ko-klan remedy extracts obtained from different extraction methods. All Ko-klan remedy extracts that maintained cell viability above 80% at a concentration of 15.6 μg/mL were selected to evaluate their inhibitory effects on NO production using the Griess reagent assay as obtained in Tables S2 and S15. At the highest tested concentration, only extracts T33 and T34 demonstrated more than 50% inhibition of NO production in LPS-stimulated RAW 264 cells. Consequently, IC_50_ values for NO inhibition were determined exclusively for these two extracts. The IC_50_ values of T33 and T34 were 16.0 ± 5.1 and 18.8 ± 1.1 μg/mL, respectively, and not significantly different from the IC_50_ of the positive control, L-NAME (20.8 ± 0.9 μg/mL), as illustrated in Figure 3(a). These findings suggested that extracts T33 and T34 showed potential as NO inhibitors to L-NAME.

In a previous study, the anti-inflammatory properties of constituents derived from the stem of *C. buchanani* were assessed utilizing RAW 264.7 macrophages and NO inhibition assays [26]. Hexane and ethyl acetate extracts demonstrated potent activity, inhibiting NO production by 84.33 ± 2.01% and 82.49 ± 0.92%, respectively, at 50 μg/mL, while maintaining > 80% cell viability [26]. Among five isolated compounds, only syringaldehyde showed significant bioactivity with an IC_50_ of 49.07 ± 1.69 μM, comparable to indomethacin (39.21 ± 1.51 μM) [26]. The Ko-klan remedy formulation-3 provided high inhibition of NO production over formulation-1 and formulation-2.

### 3.6. LOX Inhibitory Activity of Ko-Klan Remedy Extracts

COX-2 and 5-LOX are key arachidonic acid–metabolizing enzymes with interconnected pathways that amplify inflammation [33]. In this study, COX-2 expression was analyzed by qPCR, and 5-LOX activity was measured to assess the inhibitory effects of Ko-klan remedy extracts. The LOX assay measures the activity of LOX enzymes, which catalyze the oxidation of unsaturated fatty acids like sodium linoleate to produce hydroperoxides—key mediators of inflammation. The inhibition of LOX activity suggested potential anti-inflammatory properties in vitro, with results expressed as IC_50_ values. The ethanolic extracts of the Ko-klan remedy demonstrated promising anti-inflammatory activity based on LOX inhibition (Figure 3(b), Table S2 and S16).

The IC_50_ values for formulation-1 ranged from 368 to > 600 μg/mL, with MAE enhancing LOX inhibition compared to the other extraction methods. Formulation-2 showed similar activity, with IC_50_ values between 373 and 503 μg/mL. Formulation-3 exhibited stronger inhibition, with IC_50_ values as low as 62.7 μg/mL. The MAE extract of formulation-3 (T35) recorded a higher IC_50_ of 140 μg/mL, but still demonstrated superior anti-inflammatory potential relative to the other ethanolic extracts.

These findings correlated enhanced LOX inhibition with high phenolic and flavonoid contents derived from *C. sappan*, *P. interruptum*, and *C. buchananii* present in formulation-3 [34–37]. *C. buchanani* 50% ethanolic extract exhibited inhibitory activity against 5-LOX pathways [31]. In calcium ionophore A23187-stimulated rat peritoneal leukocytes, the extract inhibited 5-LOX-mediated leukotriene B4 (LTB4) production in a dose-dependent manner, with maximum inhibition at 800 μg/mL comparable to the reference standard AA 861 [31]. The extract target of the COX and 5-LOX pathways differentiates it from traditional NSAIDs, offering a more comprehensive anti-inflammatory effect [31]. However, the specific active compounds responsible for LOX inhibition of formulation-3 within these plants remain unidentified.

### 3.7. Structural Characterization of the Chemical Constituents in Ko-Klan Remedy Extracts

**Figure 4:**
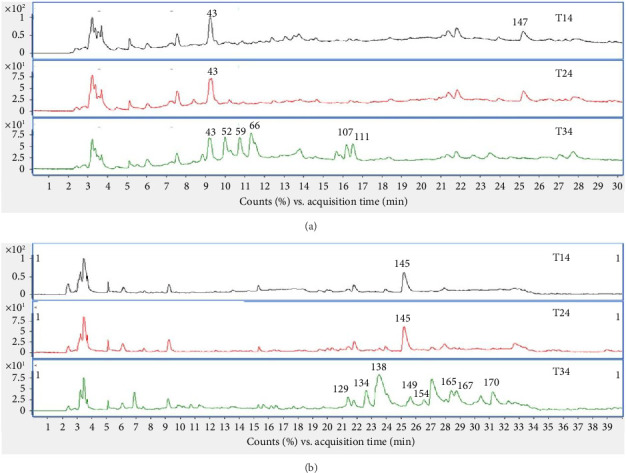
MS chromatograms of the ethanolic extracts of the traditional Thai remedy. (a) Total ion chromatograms of the crude extracts from the T14, T24, and T34 formulations, analyzed in negative ion mode (−ESI). The numbers correspond to the different compounds identified in the T3 extract, as detailed in. (b) Total ion chromatograms of the crude extracts from the T1, T2, and T3 formulations, analyzed in positive ion mode (+ESI). The numbers correspond to the different compounds identified in the T3 extract.

A total of 175 compounds were identified in the crude Ko-klan remedy extracts from formulations-1 (T14), -2 (T24), and -3 (T34). Chromatographic peaks were observed in formulation 3 extracts, with retention times between 9 and 17 min in the negative ion mode (Figure 4(a)). These peaks corresponded to homoisoflavanones (such as 3-deoxysappanone B, Sappanone A, and Sappanone B), previously reported in the heartwood of *C. sappan* (CS) [38, 39]. In the positive ion mode (Figure 4(b)), peaks detected between 21 and 32 min such as piperyline, dihydropiperlonguminine, piperlonguminine, piperettine, piperidine, dipiperamide E, and pipernonaline were derived from *P. ribesoides*. Formulation-3 also exhibited the highest bioactivity. A comprehensive list of the contained compounds is provided in the supporting information (Tables S8 and S9).

### 3.8. Molecular Docking

The 5-LOX enzyme complexed with inhibitor NDGA was used for the macromolecular docking experiment. The co-crystallized substrate was removed before modeling. The structure of 5-LOX was completed by modeling the missing residues, including residues 1–22, 59–61, 188–232, 312–321, and 434–447, to the structure using AlphaFold3. These residues are important for docking because they are located near the active site. Thus, these residues need to be completed before prediction. The completed structure is depicted in Figures S1 and S2. The docking protocol was validated by redocking co-crystallized ligand NDGA into the binding site. The results showed an RMSD value of 0.854 Å between the docked and co-crystallized structures indicating a good docking protocol. The alignment between these structures also showed a similar binding mode within the binding site. The 3D ligand structure was generated by Gaussview6 software, followed by protonation state correction at pH 8 due to the presence of the hydroxy group on the aromatic ring with various protonation states. The structure was subjected to energy minimization using the DFT calculation to avoid steric clashes.

**Figure 5:**
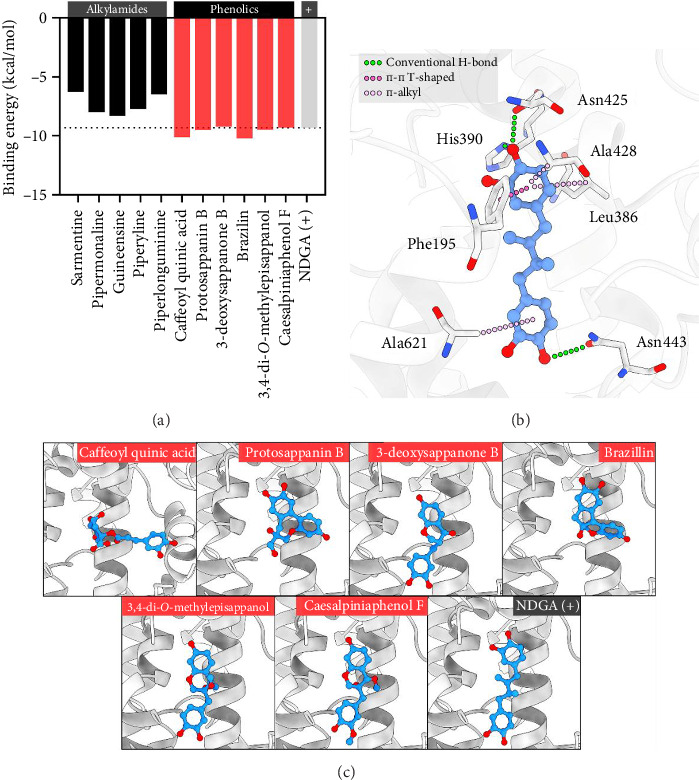
(a) Binding energy of potential compounds at the active site of 5-LOX. (b) Binding orientations of potential compounds at the active site of 5-LOX. (c) Protein-ligand interaction of co-crystallized inhibitor NDGA at the active site of 5-LOX.

The third formulation of Ko-klan showed high inhibition of LOX activity from the soybean LOX inhibition assay. The potential compounds in the third formulation, according to the MS chromatogram, predicting the binding orientations on the binding site of 5-LOX, included caffeoyl quinic acid, protosappanin B, 3-deoxysappanone B, sarmentine, pipermonaline, guineensine, piperyline, piperlonguminine, brazilin, 3,4-di-*O*-methylepisappanol, and caesalpiniaphenol F. The results are shown in Table S10 and Figure 5(a). The potential compounds were classified as alkylamide and phenolic derivatives, showing binding energies in the range −10.24 to −6.26 kcal/mol. The positive inhibitor NDGA had a binding energy of −9.33 kcal/mol. The phenolic compounds showed similar binding ability to NDGA, and higher than the alkylamides. The caffeoyl quinic acid and brazilin showed excellent binding energies than the other potential compounds, highlighting their ability to inhibit 5-LOX.

To gain insight into the atomistic interactions, the binding poses of potential compounds at the active site of 5-LOX were analyzed as depicted in Figures 5(b) and 5(c). Most of the potential compounds aligned their structure similarly to NDGA. The compounds favorably aligned their polar portions, such as pyrrolidinyl, benzo[*d*][1,3]dioxolyl, catecholyl, chromonyl, or phenolic groups to the same position of the catecholyl portion of NDGA. However, the compounds possessed catecholyl moiety. The 1,2-dihydroxybenzenyl group demonstrated stronger binding ability than the other compounds, highlighting the crucial role of this portion in binding with the active site of 5-LOX.

The binding pose of NDGA was analyzed to highlight the role of the catecholyl portion. The results suggested that the *p*-hydroxy of the catecholyl group contributed to hydrogen bonding with His390, Asn425, and Asn443. This interaction was only observed in the *p*-hydroxy group and not in the adjacent *m*-hydroxy group. Thus, the monohydroxylated aromatic ring, a benzene ring with only one hydroxy group, accommodated the same site of catecholyl group, as observed in the case of 3-deoxysappanone B, 3,4-di-*O*-methylepisappanol, and caesalpiniaphenol F. The aromatic ring of the catecholyl portion also interacted with 5-LOX through π-interactions, such as Phe195, Leu386, Ala428, and Ala621. These particular interactions were observed in all the phenolic compounds, except for caffeoyl quinic acid, which aligned the catecholyl group to another pocket.

## 4. Conclusions

This study presented a scientific validation for the traditional use of the Ko-klan remedy in relieving musculoskeletal pain by integrating phytochemical profiling, antioxidant assays, anti-inflammatory evaluations, and molecular docking. The findings indicated that the Ko-klan extracts, particularly formulation-3, possess pharmacologically relevant activities that align with their ethnomedicinal applications. These activities are associated with phenolic- and flavonoid-rich constituents that modulate oxidative stress and inflammatory mediators, with several compounds emerging as potential bioactive markers. However, the evidence is currently restricted to in vitro cellular assays and computational modeling. The results provide mechanistic insights but cannot confirm clinical efficacy. Future research should therefore focus on in vivo validation, dose-response relationships, safety assessments, and the development of standardized extraction and quality control parameters to advance the Ko-klan remedy toward therapeutic application and regulatory acceptance.

## Figures and Tables

**Table 1:** Ko-klan remedy formulations according to the national herbal medicine database (2021).

Herb	Formulation-1 (g)	Formulation-2 (g)	Formulation-3 (g)
*Mallotus repandus* (Rottler) Müll.Arg.	25	50	20
*Elephantopus scaber* L	25	25	10
*Aegle marmelos* (L.) Corrêa	25	15	—
*Rhinacanthus nasutus* (L.) Kurz	25	15	10
*Cryptolepis buchananii* Roem. and Schult	—	—	20
*Piper interruptum* Opiz	—	—	20
*Caesalpinia sappan* L.	—	—	20
Total	100	105	100

**Table 2:** Extraction methods for the Ko-klan remedies.

Extraction code^a^	Ko-klan formulation	Method	Solvent	Time
T11	First formulation	Decoction	DI-water	30 min
T12	First formulation	Decoction	EtOH	30 min
T13	First formulation	Maceration	EtOH	24 h
T14	First formulation	UAE, 30°C–40°C	EtOH	30 min
T15	First formulation	MAE, 500 W	EtOH	30 min
T22	Second formulation	Decoction	EtOH	30 min
T23	Second formulation	Maceration	EtOH	24 h
T24	Second formulation	UAE, 30°C–40°C	EtOH	30 min
T25	Second formulation	MAE, 500 W	EtOH	30 min
T32	Third formulation	Decoction	EtOH	30 min
T33	Third formulation	Maceration	EtOH	24 h
T34	Third formulation	UAE, 30°C–40°C	EtOH	30 min
T35	Third formulation	MAE, 500 W	EtOH	30 min

^a^Extraction codes: T1x = Ko-klan formulation 1, T2x = formulation 2, T3x = formulation 3 (x denotes the extraction method).; Extraction methods: T11 = decoction in deionized water (DI); Tx2 = decoction in EtOH; Tx3 = maceration in EtOH; Tx4 = ultrasound-assisted extraction (UAE) in EtOH; Tx5 = microwave-assisted extraction (MAE) in EtOH.

**Table 3:** Percentage yield, total phenolic and flavonoid contents, and antioxidant activities of Ko-klan remedy extracts.

Extract	Yield (%)	Total phenolics (g GAE/g)	Total flavonoids (mg QE/g)	DPPH (IC_50_, μg/mL) × 10^2^	ABTS (IC_50_, μg/mL) ×10^2^	FRAP (mgFe^2+^/g) ×10^2^
T11	11.2	0.454 ± 0.017	1.62 ± 0.21	1.15 ± 0.01	1.60 ± 0.05	0.207 ± 0.011
T12	4.89	0.468 ± 0.025	4.30 ± 0.35	1.75 ± 0.01	1.50 ± 0.02	0.685 ± 0.085
T13	4.41	0.413 ± 0.038	2.76 ± 0.30	2.36 ± 0.22	1.39 ± 0.09	0.850 ± 0.007
T14	1.95	0.349 ± 0.023	2.06 ± 0.12	2.38 ± 0.07	2.46 ± 0.01	1.14 ± 0.02
T15	7.47	0.597 ± 0.031	4.80 ± 0.74	0.942 ± 0.064	1.20 ± 0.07	0.739 ± 0.005
T22	4.58	0.455 ± 0.026	3.63 ± 0.35	1.08 ± 0.05	1.17 ± 0.15	0.934 ± 0.058
T23	4.32	0.365 ± 0.006	2.63 ± 0.06	1.10 ± 0.01	1.41 ± 0.13	0.912 ± 0.027
T24	2.78	0.397 ± 0.011	3.20 ± 0.36	0.912 ± 0.025	1.43 ± 0.05	1.10 ± 0.06
T25	3.91	0.467 ± 0.017	7.30 ± 0.92	1.95 ± 0.02	1.55 ± 0.05	0.996 ± 0.003
T32	3.56	1.08 ± 0.02	2.57 ± 0.81	0.617 ± 0.025	0.942 ± 0.004	1.43 ± 0.051
T33	2.86	0.860 ± 0.051	1.96 ± 0.04	0.655 ± 0.059	0.981 ± 0.014	1.60 ± 0.06
T34	1.27	0.905 ± 0.058	6.41 ± 0.96	0.518 ± 0.062	0.977 ± 0.003	1.23 ± 0.06
T35	3.89	1.08 ± 0.03	5.14 ± 1.46	0.757 ± 0.011	0.923 ± 0.004	1.65 ± 0.01
Trolox	—	—	—	0.104 ± 0.001	0.089 ± 0.001	0.765 ± 0.150

## Data Availability

Data will be made available upon reasonable request to the corresponding author.

## Supporting Information

Figure S1: 5-LOX structure refinement and docking protocol validation. Figure S2: 2-Dimensional plots of protein-ligand interactions of potential compounds within the active site of 5-LOX. Table S1: Cell viability of various extracts (T11–T35) at different concentrations. Table S2: Inhibition of NO production in lipopolysaccharide-induced RAW264.7 cells and LOX activity. Table S3: Primer sequences and conditions for qRT-PCR. Table S4: Comparative TNF-α gene expression in lipopolysaccharide-induced RAW 264 cells. Table S5: Comparative iNOS gene expression in lipopolysaccharide-induced RAW 264 cells. Table S6: Comparative IL-1β gene expression in lipopolysaccharide-induced RAW 264 cells. Table S7: Comparative COX-2 gene expression in lipopolysaccharide-induced RAW 264 cells. Table S8: Tentative identification of the chemical constituents in Ko-klan remedy formulation-1 (T1), -2 (T2), and -3 (T3). Table S9: Compounds identified in the Ko-klan remedy formulation-3 (T3), which differ from those found in formulations-1 (T1) and -2 (T2). Table S10: Molecular docking results between potential compounds and 5-LOX. Table S11: *p* values obtained from statistical analyses (one-way ANOVA with Tukey's post hoc test) comparing iNOS mRNA expressions of Ko-klan remedy extracts. Table S12: *p* values obtained from statistical analyses (one-way ANOVA with Tukey's post hoc test) comparing TNF-α mRNA expressions of Ko-klan remedy extracts. Table S13: *p* values obtained from statistical analyses (one-way ANOVA with Tukey's post hoc test) comparing COX-2 mRNA expressions of Ko-klan remedy extracts. Table S14: *p* values obtained from statistical analyses (one-way ANOVA with Tukey's post hoc test) comparing IL-1β mRNA expressions of Ko-klan remedy extracts. Table S15: *p* values obtained from statistical analyses (one-way ANOVA with Tukey's post hoc test) comparing IC_50_ values of nitric oxide (NO) inhibition of Ko-klan remedy extracts. Table S16: *p* values obtained from statistical analyses (one-way ANOVA with Tukey's post hoc test) comparing IC_50_ values of LOX inhibition of Ko-klan remedy extracts.
