# Supplementary material for: Potential of Ko-Klan Traditional Thai Remedy for Evaluation of Antioxidant and RT-PCR Anti-Inflammatory Activities
Source: Scientifica (Cairo). 2025 Nov 24;2025:4361994. doi: 10.1155/sci5/4361994 (PMC12668843; doi:10.1155/sci5/4361994)
Supplement: Supporting Information — Additional supporting information can be found online in the Supporting Information section. [file 4361994.f1.docx]

**Supporting information**

Potential of Ko-klan Traditional Thai Remedy for Evaluation of Antioxidant and RT-PCR Anti-inflammatory Activities

Sutthichat Kerdphon^a,b,c,d^, Pariya Atawong^e^, Sukanya Reanpang^e^, Phanupong Changtor^f^, Nopawit Khamto^g^, Gorawit Yusakul^e,h^, Nitra Nuengchamnong^i^, Kittisak Buddhachat^f^, Jira Jongcharoenkamol^b,c,d,e*^

^a^*Department of Chemistry, Faculty of Science, Naresuan University, Phitsanulok 65000, Thailand.*

^b^*Center of Excellence for Innovation in Chemistry (PERCH-CIC), Naresuan University, Phitsanulok 65000, Thailand.*

^c^*Center of Excellence for Innovation and Technology for Detection and Advanced Materials, Naresuan University, Phitsanulok 65000, Thailand.*

^d^*Center of Excellence for Natural Health Product Innovation, Naresuan University, Phitsanulok 65000, Thailand.*

^e^*Department of Pharmaceutical Chemistry and Pharmacognosy, Faculty of Pharmaceutical Science, Naresuan University, Phitsanulok 65000, Thailand.*

^f^*Department of Biology, Faculty of Science, Naresuan University, Phitsanulok 65000, Thailand.*

^g^*Department of Biochemistry, Faculty of Medical Science, Naresuan University, Phitsanulok 65000, Thailand.*

^h^*Research and Innovation Cluster for Natural Health Products, Naresuan University, Phitsanulok 65000, Thailand.*

^i^*Science Lab Centre, Faculty of Science, Faculty of Science, Naresuan University, Phitsanulok 65000, Thailand.*

*Corresponding author. Tel.: +66 (0)55961-870. E-mail address: Jiraj@nu.ac.th


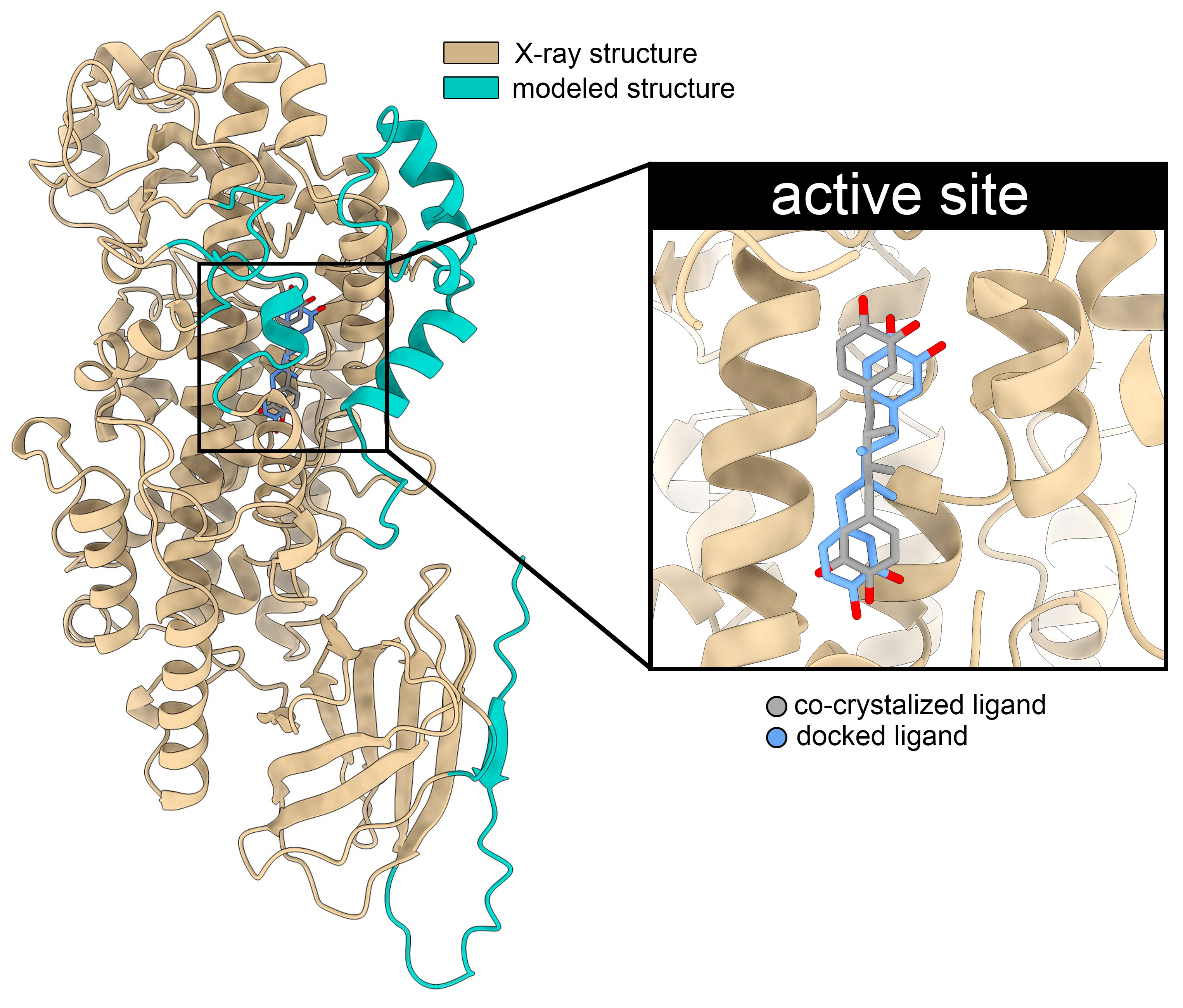


**Fig. S1** 5-LOX structure refinement and the docking protocol validation


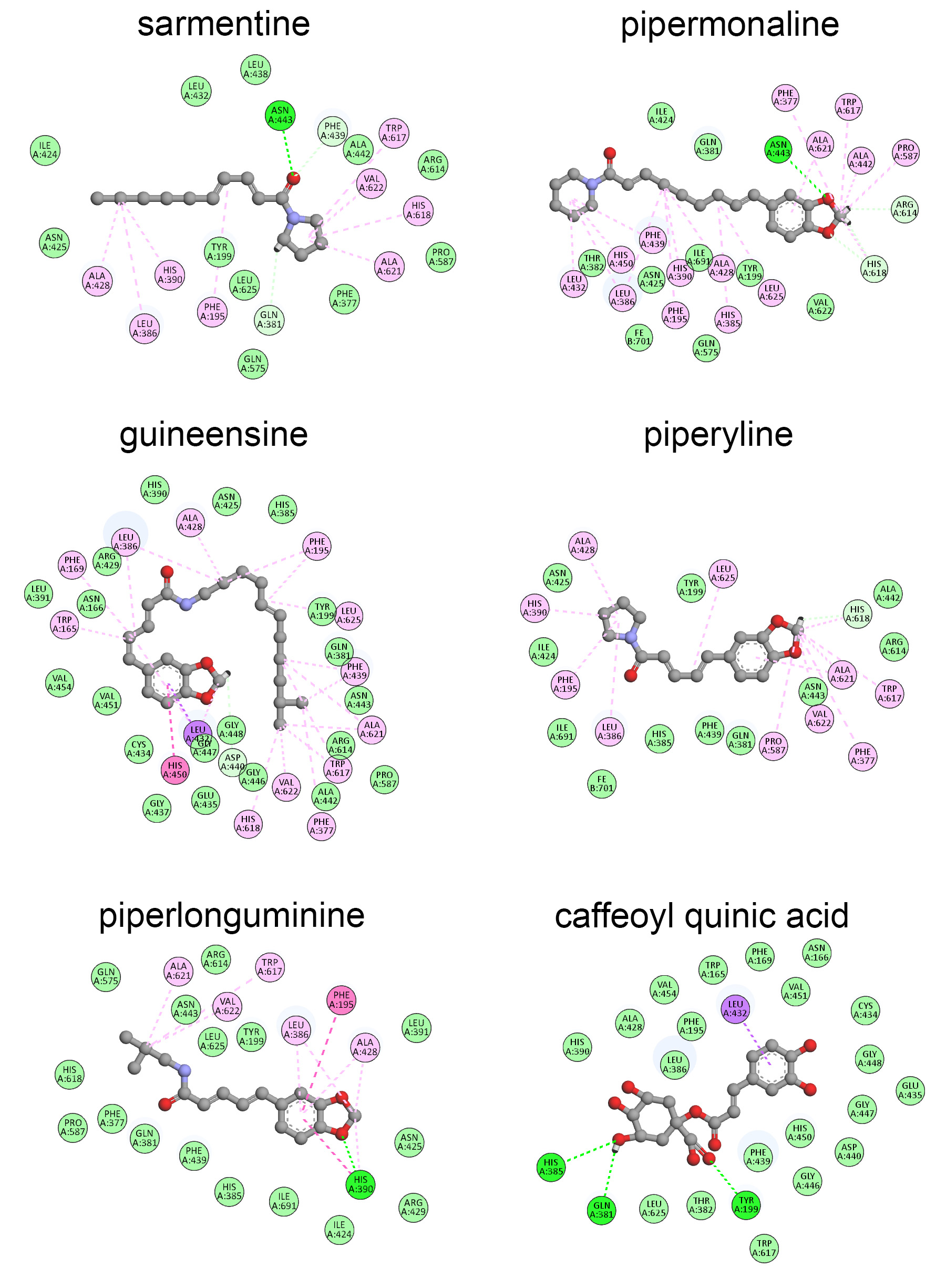


**Fig. S2** 2-Dimensional plots of protein-ligand interactions of potential compounds within active site of 5-LOX


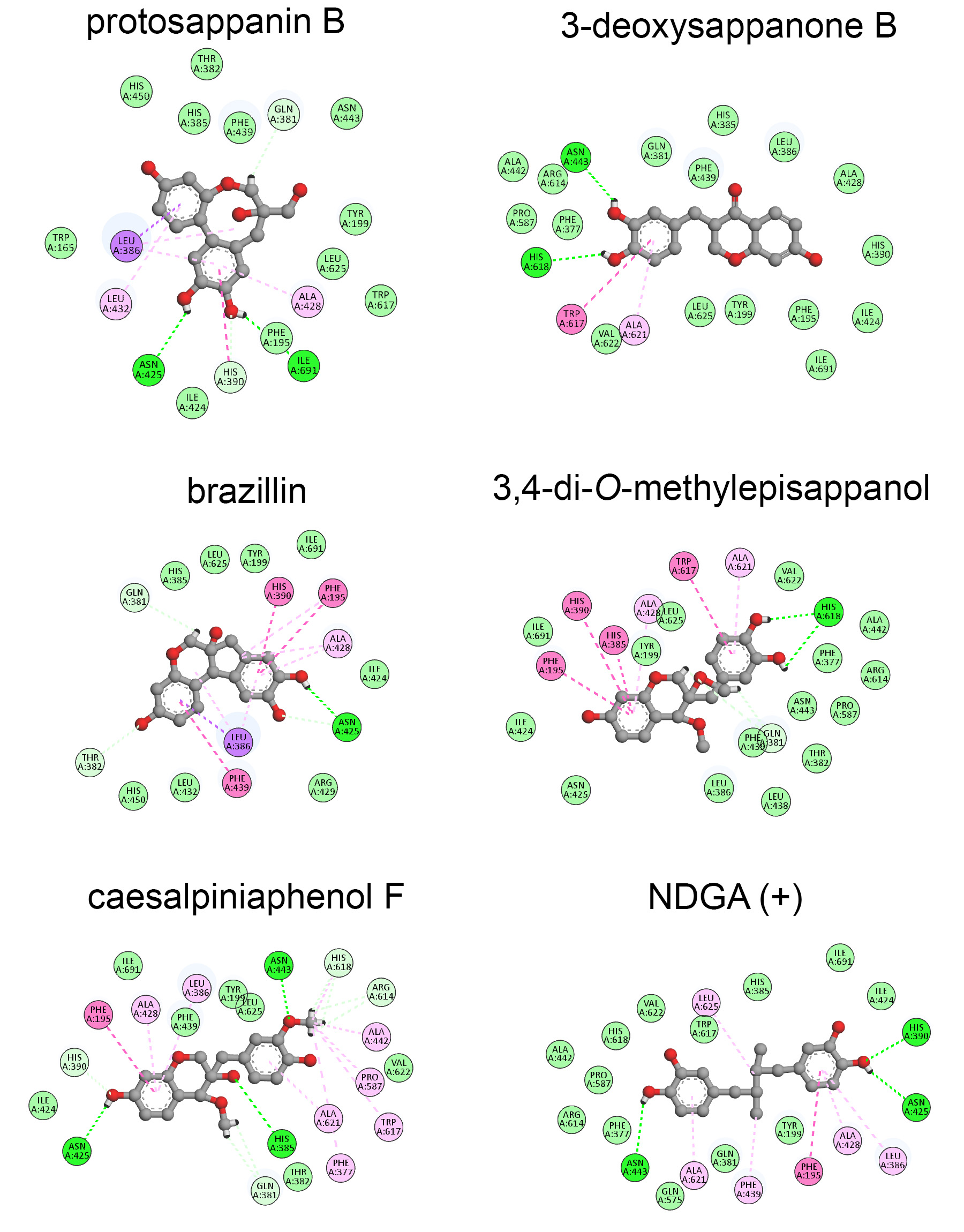


**Fig. S2** 2-Dimensional plots of protein-ligand interactions of potential compounds within active site of 5-LOX

**Table S1** Cell viability of various extracts (T11–T35) at different concentrations

| **Extract** | **Cell viability (%) of extracts at different concentration (μg/mL)** | | | | | |
| --- | --- | --- | --- | --- | --- | --- |
|  | **62.5** | **31.2** | **15.6** | **7.81** | **3.91** | **1.95** |
| T11 | n/a | 71.3±1.9 | 80.7±0.4 | 92.5±1.3 | 96.8±3.2 | 98.2±1.0 |
| T12 | n/a | 59.1±0.8 | 53.2±3.8 | 56.9±4.8 | 54.0±2.4 | 69.2±7.0 |
| T13 | 66.7±1.8 | 59.5±2.7 | 98.4±4.9 | 102±4 | 101±4 | n/a. |
| T14 | 65.0±3.5 | 59.2±4.0 | 105±4 | 104±3 | 104±4 | n/a |
| T15 | n/a | n/a | 40.8±2.1 | 45.9±2.2 | 45.8±3.2 | 47.6±3.4 |
| T22 | n/a | 50.1±2.0 | 45.5±0.6 | 42.1±0.6 | 44.8±2.6 | 49.2±2.9 |
| T25 | n/a | n/a | 46.0±0.1 | 50.3±1.3 | 50.3±4.0 | 53.3±4.1 |
| T32 | n/a | 71.4±9.6 | 83.9±12.3 | 88.0±9.0 | 81.9±14.7 | 100±4 |
| T33 | n/a | 89.6±1.8 | 103±3 | 103±4 | 102±7 | 98.8±6.3 |
| T34 | n/a | 102±6 | 103±8 | 100±10 | 97.2±7.7 | 93.5±7.6 |
| T35 | n/a | n/a | 88.0±5 | 91.9±5.6 | 99.7±5.6 | 99.1±7.0 |

T23 and T24 obtained cell viability less than 40% at concentration >1.95 μg/ml., n/a: The extracts were not tested for cell viability to RAW264 cell.

**Table S2** Inhibition of nitric oxide production in lipopolysaccharide-induced RAW264.7 cell and lipoxygenase activity

| **Extracts** | **Inhibition of nitric oxide production (IC_50_, μg/mL)** | **Lipoxygenase inhibition activity (IC_50_, μg/mL)** |
| --- | --- | --- |
| T11 | > 15.6 | > 600 |
| T12 | n/a | 545±21 |
| T13 | > 15.6 | 586±1 |
| T14 | > 15.6 | 528±10 |
| T15 | n/a | 368±4 |
| T22 | n/a | 503±16 |
| T23 | n/a | 465±21 |
| T24 | n/a | 373±4 |
| T25 | n/a | 429±6 |
| T32 | > 15.6 | 79.1±2.6 |
| T33 | 16.0±5.1 | 94.0±8.5 |
| T34 | 18.8±1.1 | 62.7±5.3 |
| T35 | > 15.6 | 140±9 |
| L-NAME | 20.8±0.9 | - |
| NDGA | - | 1.78±0.16 |

n/a: The extracts were not tested for inhibition of nitric oxide production due to cytotoxicity to RAW264 cell

**Table S3** Primer sequences and conditions for qRT-PCR

| **Genes** | **Primer sequence (5’-3’)** |
| --- | --- |
| TNF-*α* | Forward: ATG AGC ACA GAA AGC ATG ATC  Reverse: TAC AAG CTT GTC ACT CGA ATT |
| COX-2 | Forward: AGA AGG AAA TGG CTG CAG AA  Reverse: GCT CGG CTT CCA GTA TTG AG |
| iNOS | Forward: GCA GAA TGT GAC CAT GG  Reverse: ACA ACC TTG GTG TTG AAG GC |
| IL1-*β* | Forward: CTC GTG CTG TCG GAC CCA TAT  Reverse: TTG AAG ACA AAC CGC TTT TCCA |
| GAPDH | Forward: TTC ACC ACC ATG GAG AAG GC  Reverse: GGC ATG GAC TGT GGT CAT GA |

**Table S4** Comparative TNF-*α* gene expression in lipopolysaccharide-induced RAW 264 cells

| Treatment | Relative gene expression (%) |
| --- | --- |
| T11 (15.6 μg/mL) | 96.4±7.4 |
| T13 (15.6 μg/mL) | 89.6±8.8 |
| T14 (15.6 μg/mL) | 91.5±0.9 |
| T32 (15.6 μg/mL) | 128±4 |
| T33 (15.6 μg/mL) | 103±7 |
| T34 (15.6 μg/mL) | 84.6±1.3 |
| T35 (15.6 μg/mL) | 58.6±2.2 |
| Dexamethasone (31.2 μM) | 53.5±3.5 |
| Negative control | 100±3 |
| Medium control | 6.28±1.7 |

**Table S5** Comparative iNOS gene expression in lipopolysaccharide-induced RAW 264 cells

| Treatment | Relative gene expression (%) |
| --- | --- |
| T11 (15.6 μg/mL) | 64.2±0.2 |
| T13 (15.6 μg/mL) | 40.8±5.8 |
| T14 (15.6 μg/mL) | 60.2±2.8 |
| T32 (15.6 μg/mL) | 89.7±1.4 |
| T33 (15.6 μg/mL) | 74.2±1.0 |
| T34 (15.6 μg/mL) | 85.8±0.9 |
| T35 (15.6 μg/mL) | 49.6±6.0 |
| Dexamethasone (31.2 μM) | 39.0±2.2 |
| Negative control | 100±6 |
| Medium control | 1.89±0.1 |

**Table S6** Comparative IL-1*β* gene expression in lipopolysaccharide-induced RAW 264 cells

| Treatment | Relative gene expression (%) |
| --- | --- |
| T11 (15.6 mg/mL) | 65.3±15.9 |
| T13 (15.6 mg/mL) | 131±18 |
| T14 (15.6 mg/mL) | 92.7±1.5 |
| T32 (15.6 mg/mL) | 93.4±11.3 |
| T33 (15.6 mg/mL) | 60.7±2.2 |
| T34 (15.6 mg/mL) | 101±3 |
| T35 (15.6 mg/mL) | 45.3±2.2 |
| Dexamethasone (31.2 mM) | 7.60±2.06 |
| Negative control | 95.8±1.5 |
| Medium control | 0.030±0.010 |

**Table S7** Comparative COX-2 gene expression in lipopolysaccharide-induced RAW 264 cells

| Treatment | Relative gene expression (%) |
| --- | --- |
| T11 (15.6 mg/mL) | 76.4±6.7 |
| T13 (15.6 mg/mL) | 45.2±0.0 |
| T14 (15.6 mg/mL) | 115±10 |
| T32 (15.6 mg/mL) | 206±4 |
| T33 (15.6 mg/mL) | 94.0±6.5 |
| T34 (15.6 mg/mL) | 113±11 |
| T35 (15.6 mg/mL) | 43.9±3.3 |
| Dexamethasone (31.2 mM) | 25.7±2.3 |
| Negative control | 100±5 |
| Medium control | 0.270±0.090 |

**Table S8** The tentative identification of chemical constituents in Ko-klan remedy formulation-1 (T1), -2 (T2), and -3 (T3)

| **No.** | **RT(min)** | **m/z** | **Adduct** | **MS/MS** | **Tentative Identification** | **Formula** | **Found** |
| --- | --- | --- | --- | --- | --- | --- | --- |
| 1 | 2.81 | 128.9596 | [M-H]- |  | Unidentified |  | T2 |
| 2 | 3.173 | 293.099 | [M-H]- | 203.0682,131.0460,68.9950 | N-Glycosyl-L-asparagine | C_10_ H_18_ N_2_O_8_ | T1 |
| 3 | 3.191 | 156.102 | [M+H]+ | 70.0643,68.0491 | 2-Amino-2-Norbornanecarboxylic acid | C_8_H_13_NO_2_ | T3 |
| 4 | 3.219 | 179.0562 | [M-H]- | 59.0135 | Hexose | C_6_H_12_O_6_ | T1,T2,T3 |
| 5 | 3.225 | 273.1813 | [M+H]+ | 118.0853,58.0645 | N-Octanoylglutamine | C_13_H_24_N_2_O_4_ | T3 |
| 6 | 3.244 | 118.0862 | [M+H]+ | 58.0652 | 2-Amino-3-methylbutanoic acid | C_5_ H_11_NO_2_ | T1,T2,T3 |
| 7 | 3.278 | 195.0507 | [M-H]- | 75.0079,59.0128 | Gluconic acid | C_6_H_12_O_7_ | T1 |
| 8 | 3.331 | 683.2337 | [2M-H]- | 341.1143,179.0553,89.0241 | Sucrose | C_12_H_22_O_11_ | T1,T2,T3 |
| 9 | 3.479 | 144.1012 | [M+H]+ | 102.0544,84.0805,58.0648 | 1-Aminocyclohexanecarboxylic acid | C_7_H_13_NO_2_ | T1,T2,T3 |
| 10 | 3.496 | 191.0562 | [M-H]- | 85.029 | Quinic acid | C_7_H_12_O_6_ | T1,T2,T3 |
| 11 | 3.526 | 533.1744 | [M+HCOO]- | 191.0570,85.0290 | Fucosyllactose | C_18_H_32_O_15_ | T1 |
| 12 | 3.573 | 179.0555 | [M-H]- | 75.0079 | Fructose | C_6_H_12_O_6_ | T2 |
| 13 | 3.638 | 130.0865 | [M+H]+ |  | Cycloleucine | C_6_H_11_NO_2_ | T1 |
| 14 | 3.709 | 133.0133 | [M-H]- |  | Malic acid | C_4_H_6_O_5_ | T1 |
| 15 | 4.459 | 133.0144 | [M-H]- |  | Malic acid | C_4_H_6_O_5_ | T1,T2 |
| 16 | 5.122 | 290.0896 | [M-H]- | 200.0562,128.0350 | Unidentified |  | T1,T2 |
| 17 | 5.126 | 481.068 | [M-H]- | 301.0019,275.0197,231.0130,59.0158 | Luteic glucoside | C20H18O14 | T3 |
| 18 | 5.131 | 160.1299 | [M+H]+ | 58.0603 | Unidentified |  | T1,T2,T3 |
| 19 | 5.145 | 191.0207 | [M-H]- | 111.0087,87.0085 | Citric acid | C_6_H_8_O_7_ | T2,T3 |
| 20 | 5.518 | 188.0577 | [M-H]- | 128.0350,102.0552,59.0132 | N-Acetyl-L-glutamic acid | C_7_H_11_NO_5_ | T3 |
| 21 | 5.63 | 481.0676 | [M-H]- | 301.0039,275.0218 | Luteic glucoside | C_20_H_18_O_14_ | T1,T2 |
| 22 | 6.004 | 243.0658 | [M-H]- |  | Unidentified |  | T1,T3 |
| 23 | 6.032 | 331.0705 | [M-H]- |  | Unidentified |  | T1,T3 |
| 24 | 6.04 | 117.0191 | [M-H]- | 73.0289 | Succinic acid | C_4_H_6_O_4_ | T1,T2,T3 |
| 25 | 6.048 | 331.0692 | [M-H]- | 271.0472,211.0293,169.0160,125.0238 | Gallyl glucoside | C_13_H_16_O_10_ | T2 |
| 26 | 6.094 | 269.1037 | [M+H]+ | 203.0337,135.0554 | Unidentified |  | T3 |
| 27 | 6.117 | 135.0551 | [M+H]+ | 84.0788,52.0164 | Unidentified |  | T1,T2 |
| 28 | 6.197 | 115.0039 | [M-H]- |  | Maleic Acid | C_4_H_4_O_4_ | T1,T3 |
| 29 | 6.901 | 174.1493 | [M+H]+ | 97.0641,58.0649 | Nonanoic acid, 3-amino-, (R)- | C_9_H_19_NO_2_ | T3 |
| 30 | 7.183 | 169.0151 | [M-H]- |  | Gallic acid | C_7_H_6_O_5_ | T1,T2,T3 |
| 31 | 7.269 | 313.0591 | [M-H]- | 235.0274,193.0152,151.0391,93.0354 | Norbergenin | C_13_H_14_O_9_ | T1,T3 |
| 32 | 7.302 | 328.1396 | [M+H]+ |  | Unidentified |  | T1 |
| 33 | 7.508 | 315.0713 | [M+H]+ | 279.0477,249.0403,219.0295,167.0344, 103.0543,77.0388 | Norbergenin | C_13_ H_14_ O_9_ | T1,T2,T3 |
|  | 7.545 | 313.0605 | [M-H]- | 235.0258,193.0151,93.0356 | Norbergenin | C_13_H_14_O_9_ | T1,T2,T3 |
| 34 | 8.222 | 349.073 | [M-H]- |  | Unidentified |  | T1 |
| 35 | 8.323 | 427.2085 | [M+H]+ |  | Unidentified |  | T2 |
| 36 | 9.32 | 363.0504 | [M+Cl]- | 327.0766,207.0305 | Bergenin | C_14_H_16_O_9_ | T2 |
| 37 | 8.39 | 305.0694 | [M-H]- |  | (-)-Epigallocatechin | C_15_H_14_O_7_ | T1,T2,T3 |
| 38 | 8.482 | 265.1552 | [M+H]+ | 177.0549,145.0286,117.0337, 89.0385,63.0228 | Val Phe | C_14_ H_20_N_2_O_3_ | T2 |
| 39 | 8.527 | 899.2351 | [M-H]- | 737.1861,557.1211,421.0604,125.0230 | Unidentified | C_42_H_44_O_22_ | T1 |
| 40 | 8.555 | 153.0202 | [M-H]- |  | 2,4-Dihydroxybenzoic acid | C_7_H_6_O_4_ | T3 |
| 41 | 8.842 | 353.0931 | [M-H]- | 191.0575,135.0449,85.0290 | Caffeoyl quinic acid | C_16_H_18_O_9_ | T1,T3 |
| 42 | 9.155 | 883.2398 | [2M-H]- | 721.1999,567.1285,387.0564 | 2,2'-Diphenyl-4H,4'H-[3,3'-bichromene] -4,4'-dione | C_30_H_18_O_4_ | T1 |
| 43 | 9.185 | 329.0871 | [M+H]+ | 293.0654,263.0553,233.0439,181.0495, 150.0314,131.0493,77.0388 | Bergenin | C_14_H_16_O_9_ | T1,T2,T3 |
|  | 9.216 | 327.075 | [M-H]- |  | Bergenin | C_14_H_16_O_9_ | T3 |
| 44 | 9.294 | 153.0195 | [M-H]- |  | 2,4-Dihydroxybenzoic acid | C_7_H_6_O_4_ | T3 |
| 45 | 9.491 | 633.0858 | [M-H]- | 463.0638,301.0011,169.0138 | Punicacortein B | C_27_H_22_O_18_ | T3 |
| 46 | 9.519 | 883.2399 | [2M-H]- | 721.1906,567.1230,405.0759, 271.0631,125.0224 | 2,2'-Diphenyl-4H,4'H-[3,3'-bichromene] -4,4'-dione | C_30_H_18_O_4_ | T1 |
| 47 | 9.55 | 633.0793 | [M-H]- |  | Punicacortein B | C_27_H_22_O_18_ | T1,T2 |
| 48 | 9.666 | 801.0862 | [M-H]- | 757.1033,631.0689,463.0472,300.9701 | Sanguiin H7 | C_34_H_26_O_23_ | T2,T3 |
| 49 | 9.707 | 356.1864 | [M+H]+ | 280.1077,165.0687,72.0807,58.0647 | Unidentified |  | T3 |
| 50 | 9.82 | 513.1469 | [M-H]- | 293.0863,89.0234 | Unidentified |  | T3 |
| 51 | 9.841 | 342.1704 | [M+H]+ | 265.0856,165.0693,58.0648 | Unidentified |  | T3 |
| 52 | 9.968 | 355.1031 | [M+H]+ | 163.0390,89.0386 | Caffeoyl quinic acid | C_16_H_18_O_9_ | T1,T2,T3 |
| 52 | 9.994 | 353.0917 | [M-H]- | 271.0546,191.0570,93.0324 | Caffeoyl quinic acid | C_16_H_18_O_9_ | T3 |
| 53 | 10.159 | 633.0874 | [M-H]- | 463.0639,301.0007,169.0144 | Corilagin | C_27_H_22_O_18_ | T1,T2,T3 |
| 54 | 10.235 | 342.1705 | [M+H]+ | 265.0861,163.0337,117.0337,89.0387,58.0650 | Unidentified | C_20_H_20_O_4_ | T3 |
| 55 | 10.259 | 867.2456 | [M-H]- | 777.6981,705.1971,551.1311,417.1064, 271.0610,125.0232 | diinnovanoside A | C_42_H_44_O_20_ | T1 |
| 56 | 10.263 | 303.0915 | [M-H]- | 163.0397,123.0455 | 3'-O-Methylcatechin | C_16_H_16_O_6_ | T3 |
| 57 | 10.488 | 663.1829 | [M-H]- | 627.1979,285.0800,163.0412 | Protosappanin B diglucoside | C_28_H_36_O_16_ | T4 |
| 58 | 10.625 | 501.1265 | [M-H]- | 285.0803,163.0401 | Protosappanin B glucoside | C_22_H_26_O_11_ | T5 |
| 59 | 10.723 | 322.1288 | [M+NH4]+ | 229.0492,165.0698,139.0538,115.0540, 91.0539,68.9966 | Protosappanin B | C_16_H_16_O_6_ | T3 |
|  | 10.752 | 303.0907 | [M-H]- | 231.0684,109.0285 | Protosappanin B | C_16_H_16_O_6_ | T3 |
| 60 | 10.726 | 287.0916 | [M+H]+ |  | Brazillin | C_16_H_14_O_5_ | T3 |
| 61 | 10.756 | 867.2456 | [M-H]- | 777.7116,705.1938,551.1321,417.1025, 271.0582,125.0199 | Dodegranoside B | C_42_H_44_O_20_ | T1,T2 |
| 62 | 10.892 | 449.1135 | [M-H]- | 342.0669,269.0491,125.0239 | Unidentified |  | T1,T2 |
| 63 | 11.015 | 515.1289 | [M-H]- | 353.0919,191.0561,179.0358 | Dicaffeoylquinic acid | C_25_H_24_O_12_ | T1,T2,T3 |
| 64 | 11.039 | 356.1867 | [M+H]+ | 253.0851,165.0693,137.0600,58.0653 | Unidentified |  | T3 |
| 65 | 11.226 | 333.1022 | [M-H]- |  | Methyl 2-(4,4,5'- trihydroxy-2-(methoxymethyl) biphenyl-2-yloxy) acetate | C_17_H_18_O_7_ | T3 |
| 66 | 11.303 | 287.092 | [M+H]+ | 269.0798,131.0485,103.0536,77.0382, 51.0224 | (-)-3-Deoxysappanone B | C_16_H_14_O_5_ | T3 |
|  | 11.331 | 285.0798 | [M-H]- | 163.0404,121.0292 | (-)-3-Deoxysappanone B | C_16_H_14_O_5_ | T3 |
| 67 | 11.355 | 337.0962 | [M-H]- | 191.0573,93.0336 | *p*-Coumaroylquinic acid | C_16_H_18_O_8_ | T1 |
| 68 | 11.363 | 153.0196 | [M-H]- |  | 2,4-Dihydroxybenzoic acid | C_7_H_6_O_4_ | T3 |
| 69 | 11.408 | 179.0359 | [M-H]- |  | Caffeic acid | C_9_H_8_O_4_ | T3 |
| 70 | 11.413 | 433.0488 | [M-H]- | 299.9965 | Ellagic acid arabinoside | C_19_H_14_O_12_ | T1,T2,T3 |
| 71 | 11.447 | 867.2462 | [M-H]- | 705.1951,579.1593,433.0470,255.0757, 125.0194 | Sanshiside D | C_39_H_48_O_22_ | T1,T2 |
| 72 | 11.515 | 269.0812 | [M+H]+ | 165.0701,115.0541,77.0388,51.0228 | (E)-7-Hydroxy-3-(4-hydroxybenzylidene)chroman-4- one | C_16_H_12_O_4_ | T3 |
| 73 | 11.533 | 333.102 | [M-H]- |  | Methyl 2-(4,4,5'- trihydroxy-2-(methoxymethyl) biphenyl-2-yloxy) acetate | C_17_H_18_O_7_ | T3 |
| 74 | 11.541 | 303.0931 | [M-H]- | 163.0401,91.0511 | 4'-*O*-Methylcatechin | C_16_H_16_O_6_ | T3 |
| 75 | 11.646 | 479.0917 | [M-H]- | 327.0874,249.0415,169.0138,125.0235 | 4-*O*-Galloylbergenin | C_21_H_20_O_13_ | T1,T2,T3 |
| 76 | 11.729 | 447.0643 | [M-H]- | 299.9943,160.8438 | Ellagic acid 2-rhamnoside | C_20_H_16_O_12_ | T1,T2,T3 |
| 77 | 11.827 | 287.0951 | [M-H]- |  | 3-Deoxyepisappanol | C_16_H_16_O_5_ | T3 |
| 78 | 11.837 | 559.1568 | [M-H]- | 353.0926,179.0362 | Unidentified |  | T3 |
| 79 | 11.976 | 177.0204 | [M-H]- | 121.0291,92.0250,64.0007 | Unidentified |  | T3 |
| 80 | 12.112 | 433.1185 | [M-H]- | 271.0627,227.0743,151.0022 | 5,7,8-Trihydroxyflavanone 7-glucoside | C_21_H_22_O_10_ | T1 |
| 81 | 12.385 | 367.1073 | [M-H]- |  | Feruloylquinic acid | C_17_H_20_O_9_ | T1 |
| 82 | 12.386 | 317.1053 | [M-H]- |  | 3 -*O*-Methylesappanol | C_17_H_18_O_6_ | T3 |
| 83 | 12.479 | 851.2476 | [M-H]- | 689.2050,563.1640,401.1056,255.0712, 125.0224 | Peracetylmacrophylloside D | C_39_H_48_O_21_ | T2 |
| 84 | 12.544 | 675.1816 | [M-H]- | 639.2056,517.1591,415.1367,301.0946, 223.0623 | Unidentified |  | T3 |
| 85 | 12.671 | 317.0705 | [M-H]- | 109.0283 | Unidentified |  | T3 |
| 86 | 12.753 | 301.0029 | [M-H]- | 229.0202,137.0206 | Ellagic acid | C_14_H_6_O_8_ | T3 |
| 87 | 12.818 | 285.0795 | [M-H]- | 267.0709,199.0825,109.0290 | Brazillin | C_16_H_14_O_5_ | T3 |
| 88 | 13.012 | 515.1297 | [M-H]- | 353.0921,255.0684,173.0463,93.0334 | Dicaffeoylquinic acid | C_25_H_24_O_12_ | T1,T2,T3 |
| 89 | 13.427 | 285.0759 | [M+H]+ | 257.0796,221.0596,165.0693,147.0438, 102.0460,51.0225 | Sappanone A | C_16_H_12_O_5_ | T3 |
|  | 13.453 | 283.0647 | [M-H]- |  | Sappanone A | C_16_H_12_O_5_ | T3 |
| 90 | 13.478 | 515.1293 | [M-H]- | 353.0940,191.0574 | Dicaffeoylquinic acid | C_25_H_24_O_12_ | T1,T2,T3 |
| 91 | 13.601 | 462.2134 | [M+H]+ | 346.1662,128.0706,70.0651 | Trp Trp Ala | C_25_H_27_N_5_O_4_ | T1 |
| 92 | 13.712 | 315.0549 | [M-H]- |  | Caesalpiniaphenol G | C_16_H_12_O_7_ | T3 |
| 93 | 13.729 | 515.129 | [M-H]- | 353.0933,255.0690,173.0463,93.0348 | Dicaffeoylquinic acid | C_25_H_24_O_12_ | T1,T2,T3 |
| 94 | 13.838 | 301.0754 | [M-H]- | 179.0354,151.0403,109.0274,65.0389 | Sappanone B | C_16_H_14_O_6_ | T3 |
| 95 | 14.243 | 571.172 | [M-H]- | 447.1145,297.0763,123.0439 | Unidentified |  | T3 |
| 96 | 14.284 | 353.0845 | [M+Cl]- |  | 3 -*O*-Methylepisappanol | C_17_H_18_O_6_ | T3 |
| 97 | 14.403 | 469.096 | [M-H]- | 433.1244,271.0646,151.0032 | 5,7,8-Trihydroxyflavanone 7-glucoside | C_21_H_22_O_10_ | T1 |
| 98 | 14.437 | 603.1621 | [2M-H]- | 301.0756,229.0523,159.0450 | Protosappanin C | C_16_H_14_O_6_ | T3 |
| 99 | 14.598 | 187.0989 | [M-H]- | 125.0970,57.0344 | 3-Methylsuberic acid | C_9_H_16_O_4_ | T1,T2,T3 |
| 100 | 14.798 | 559.156 | [M-H]- | 397.1190,273.0843,173.0460,93.0359 | Unidentified |  | T3 |
| 101 | 15.086 | 603.1623 | [2M-H]- | 301.0763,229.0533,159.0452 | Protosappanin C | C_16_H_14_O_6_ | T3 |
| 102 | 15.251 | 585.1514 | [M-H]- | 423.0839,283.0646,163.0353 | Unidentified |  | T3 |
| 103 | 15.665 | 287.0916 | [M+H]+ | 257.0428,151.0387,108.0201,89.0382 | Sappanchalcone | C_16_H_14_O_5_ | T3 |
|  | 15.68 | 285.08 | [M-H]- | 253.0515,163.0405 | Sappanchalcone | C_16_H_14_O_5_ | T3 |
| 104 | 15.761 | 677.1594 | [M-H]- | 515.1278,353.0917,173.0462 | 3,4,5-Tricaffeoylquinic acid | C_34_H_30_O_15_ | T2 |
| 105 | 16.138 | 497.0853 | [M-H]- | 335.0799,161.0267 | Methyl 4,6-di-O-galloyl-beta- D-glucopyranoside | C_21_H_22_O_14_ | T2 |
| 106 | 16.181 | 269.081 | [M+H]+ | 176.0613,152.0613,123.0432,103.0536, 77.0380,51.0224 | (E)-7-Hydroxy-3-(4-hydroxybenzylidene)chroman-4- one | C_16_H_12_O_4_ | T3 |
| 107 | 16.202 | 367.101 | [M+Cl]- | 331.1078,285.0800,163.0408,148.0167,121.0293, 91.0550,65.0384 | Caesalpiniaphenol F | C_18_H_20_O_6_ | T3 |
| 108 | 16.203 | 285.0801 | [M-H]- | 163.0401,148.0171,121.0297,93.0346 | (-)-3-Deoxysappanone B | C_16_H_14_O_5_ | T3 |
| 109 | 16.232 | 283.0639 | [M-H]- |  | Sappanone A | C_16_H_12_O_5_ | T3 |
| 110 | 16.388 | 385.1826 | [M-H]- |  | Unidentified |  | T2 |
| 111 | 16.503 | 333.1329 | [M+H]+ | 223.0750,176.0618,152.0615,115.0540, 77.0387,51.0228 | 3 ,4- di-*O*-Methylepisappanol | C_18_H_20_O_6_ | T3 |
|  | 16.521 | 367.1011 | [M+Cl]- | 331.1222,163.0406,121.0289 | 3 ,4- di-*O*-Methylepisappanol | C_18_H_20_O_6_ | T3 |
| 112 | 16.523 | 685.2771 | [M+H]+ | 548.1913,383.1122,351.0859,231.0649, 121.0646 | Unidentified |  | T3 |
| 113 | 16.742 | 285.043 | [M-H]- | 133.0288,65.0042 | Kaempferol | C_15_H_10_O_6_ | T2 |
| 114 | 17.16 | 274.2748 | [M+H]+ | 70.0651 | C16 Sphinganine | C_16_ H_35_NO_2_ | T3 |
| 115 | 17.402 | 271.0973 | [M+H]+ |  | Imperatorin | C_16_H_14_O_4_ | T3 |
|  | 17.409 | 269.0845 | [M-H]- |  | Imperatorin | C_16_H_14_O_4_ | T3 |
| 116 | 17.95 | 269.0853 | [M-H]- |  | Alloimperatorin | C_16_H_14_O_4_ | T3 |
| 117 | 18.389 | 329.2387 | [M-H]- | 211.1341,171.1020,139.1113, 99.0812,57.0347 | 12,13,15-Trihydroxy-9E-octadecenoic acid | C_18_H_34_O_5_ | T1,T2,T3 |
| 118 | 19.329 | 379.1435 | [M-H]- | 291.0922,203.1113,159.1186,87.0455 | Unidentified |  | T1 |
| 119 | 19.425 | 280.1333 | [M+H]+ | 131.0486,103.0537,77.0382,51.0223 | Unidentified |  | T1,T2 |
| 120 | 19.606 | 187.0393 | [M+H]+ | 159.0420,131.0485,103.0539,77.0382, 51.0226 | Psoralen | C_11_H_6_O_3_ | T1,T2 |
| 121 | 19.609 | 341.1505 | [M+H]+ | 323.1392,255.0756,227.0813,183.0673, 123.0550,79.0412 | N2,N5-Dibenzoyl-L-ornithine | C_19_H_20_N_2_O_4_ | T1 |
| 122 | 19.926 | 663.4029 | [M+H]+ | 617.8592,549.2944,400.2476,323.1388, 265.0763,106.0285 | Unidentified |  | T1,T2 |
| 123 | 19.965 | 217.0497 | [M+H]+ | 174.0303,89.0381 | Unidentified |  | T1,T2 |
| 124 | 20.207 | 601.3659 | [M+Na]+ | 532.2928,377.1830,307.1441,238.0739 | 4-Ketoalloxanthin | C_40_H_50_O_3_ | T1,T2 |
| 125 | 20.915 | 347.1899 | [M-H]- | 285.1887,269.1562,135.0806 | Unidentified |  | T1 |
| 126 | 21.115 | 379.0992 | [M-H]- | 301.0038 | Unidentified |  | T1,T2 |
| 127 | 21.33 | 381.1189 | [M+Cl]- |  | Catechin Tetramethylether | C_19_H_22_O_6_ | T1,T2,T3 |
|  | 21.357 | 364.1763 | [M+NH4]+ | 213.0908,169.1011,142.0774,91.0539 | Catechin Tetramethylether | C_19_H_22_O_6_ | T1 |
| 128 | 21.356 | 259.0969 | [M+H]+ | 219.1746,115.0551,77.0384,51.0225 | Rhinacanthin A | C_15_H_14_O_4_ | T1,T2,T3 |
| 129 | 21.421 | 272.1288 | [M+H]+ | 201.0541,115.0541 | Piperyline | C_16_H_17_NO_3_ | T3 |
| 130 | 21.788 | 347.149 | [M+H]+ | 259.0949,213.0904,169.1010,141.0693, 115.0536,77.0383 | Epicatechin Tetramethylether | C_19_H_22_O_6_ | T1,T2,T3 |
| 131 | 21.812 | 259.0965 | [M+H]+ |  | Rhinacanthin A | C_15_H_14_O_4_ | T1,T2,T3 |
| 132 | 22.528 | 276.1592 | [M+H]+ | 201.0551,171.0437,135.0442,77.0387, 51.0228 | Dihydropiperlonguminine | C_16_H_21_NO_3_ | T3 |
| 133 | 22.533 | 286.1442 | [M+H]+ |  | Chavicine | C_17_H_19_NO_3_ | T3 |
| 134 | 22.663 | 274.144 | [M+H]+ | 201.0549,171.0441,115.0543,77.0389 | Piperlonguminine | C_16_H_19_NO_3_ | T3 |
| 135 | 22.656 | 523.3311 | [M+Cl]- | 487.3520,409.1854 | 11-Acetoxy-3β,6α-dihydroxy-24-methylene-9,11-seco-5α-cholesta-7,22E-dien-9-one | C_30_H_48_O_5_ | T3 |
| 136 | 23.294 | 288.1581 | [M+H]+ | 201.0544,171.0434,115.0535,89.0383 | Dihydropiperine | C17 H21 N O3 | T2,T3 |
| 137 | 23.315 | 685.367 | [M+H]+ | 537.3100,469.2486,334.1801,266.1181, 131.0493,69.0699 | Unidentified |  | T2 |
| 138 | 23.558 | 286.1445 | [M+H]+ | 201.0548,115.0543 | Piperine | C_17_H_19_NO_3_ | T2,T3 |
| 139 | 23.933 | 479.1798 | [M-H]- | 383.1019,301.0053,59.0136 | Unidentified |  | T2 |
| 140 | 23.936 | 369.1819 | [M+H]+ | 323.1389,255.0761,133.0396,79.0415, 52.0293 | Unidentified |  | T1 |
| 141 | 23.951 | 334.1808 | [M+H]+ | 266.1168,202.0773,131.0489,131.0489, 103.0540,77.0383,51.0224 | (a*S*,b*S*)-a-Ethyl-a-(4-methoxyphenyl)- b-phenyl-2-pyridineethanol | C_22_H_23_NO_2_ | T1,T2 |
| 142 | 23.964 | 386.1568 | [M-H]- | 311.1760,200.0629,61.9875 | Unidentified |  | T1,T2 |
| 143 | 24.556 | 349.2216 | [M+Cl]- | 313.2372,201.1158 | 10-Hydroxy-9-ketooctadecanoic acid | C_18_H_34_O_4_ | T3 |
| 144 | 24.565 | 302.1754 | [M+H]+ | 167.1307,135.0443,103.0545,77.0388 | (6E)-7-(2H-1,3-Benzodioxol-5-YL) -1-(pyrrolidin-1-YL)hept-6-EN-1-one | C_18_H_23_NO_3_ | T3 |
| 145 | 25.19 | 203.0343 | [M+H]+ | 147.0442,129.0338,91.0541,75.0236 | Xanthotoxol | C_11_H_6_O_4_ | T1,T2,T3 |
| 146 | 25.192 | 271.097 | [M+H]+ | 185.0217,171.0439,147.0440,115.0538, 91.0537,77.0384 | 4,4 -Dihydroxy-2 -methoxychalcone | C_16_H_14_O_4_ | T3 |
| 147 | 25.276 | 347.2021 | [M+Cl]- |  | 9-Octadecenedioic acid, (9Z)- | C_18_H_32_O_4_ | T1,T2 |
| 148 | 25.445 | 312.1595 | [M+H]+ | 227.0695,197.0591,164.1071,135.0440, 77.0387 | Piperettine | C_19_H_21_NO_3_ | T3 |
| 149 | 25.649 | 314.1751 | [M+H]+ | 227.0700,169.0648,135.042,77.0387 | Piperidine | C_19_H_23_NO_3_ | T3 |
| 150 | 25.857 | 339.206 | [M-H]- | 183.0094 | Unidentified |  | T2 |
| 151 | 26.34 | 293.1892 | [M+Cl]- | 195.1432,96.9616 | 15-Hydroxy-pentadecanoic acid | C_15_H_30_O_3_ | T3 |
| 152 | 26.539 | 541.1959 | [M-H]- |  | Unidentified |  | T2 |
| 153 | 26.554 | 345.1867 | [M+Cl]- | 291.2009,71.0519 | 11,14-Dihydroxyoctadeca-9Z-en-12-ynoic acid | C_18_H_30_O_4_ | T1,T2 |
| 154 | 26.571 | 222.1855 | [M+H]+ | 194.1899,150.0904,124.0754,81.0334 | Sarmentine | C_14_H_23_NO | T3 |
| 155 | 26.632 | 586.3882 | [M+Cl]- | 550.4071,341.2561,225.1519 | Unidentified |  | T3 |
| 156 | 27.019 | 441.2008 | [M-H]- | 409.1688,354.1490,181.0879,137.0951 | 1,2-Dihexanoyl-sn-glycero-3-phospho-(1'-sn-glycerol) | C_18_H_35_O_10_P | T1,T2,T3 |
| 157 | 27.145 | 224.2008 | [M+H]+ | 168.1380,123.1164,81.0332 | 2,4-Decadienamide | C14H25NO | T2,T3 |
| 158 | 27.358 | 365.1691 | [M-H]- |  | Unidentified |  | T1,T2 |
| 159 | 27.709 | 600.4043 | [M+Cl]- | 564.4200,355.2771,242.1904 | Unidentified |  | T3 |
| 160 | 27.764 | 295.232 | [M-H]- | 277.2244,233.2263,195.1403,171.1012, 123.1153,98.9558 | 17-Hydroxylinoleic acid | C_18_H_32_O_3_ | T2,T3 |
| 161 | 27.956 | 307.1449 | [M+H]+ | 238.0742,154.0652,79.0419,52.0307 | Unidentified |  | T1,T2 |
| 162 | 28.057 | 347.2031 | [M+Cl]- | 293.2109,113.0969 | 5S,8R-Dihydroxy-9Z,12Z-octadecadienoic acid | C_18_H_32_O_4_ | T1 |
| 163 | 28.61 | 295.2272 | [M+H]+ |  | 9-HOTE | C_18_H_30_O_3_ | T2 |
| 164 | 28.048 | 571.2816 | [M+H]+ | 486.1921,373.1071,310.1441,192.1020, 86.0965 | Dipiperamide E | C_34_H_38_N_2_O_6_ | T3 |
| 165 | 28.397 | 342.2064 | [M+H]+ | 229.1220,135.0442,77.0387 | Pipernonaline | C_21_H_27_NO_3_ | T3 |
| 166 | 28.962 | 295.2274 | [M+H]+ |  | 9-HOTE | C_18_H_30_O_3_ | T2 |
| 167 | 28.788 | 236.2012 | [M+H]+ | 53.0387 | Piperidide | C_15_H_25_NO | T2,T3 |
| 168 | 29.605 | 349.2176 | [M+Cl]- | 295.2285 | Octadecanedioic acid | C_18_H_34_O_4_ | T1,T2 |
| 169 | 30.439 | 252.2327 | [M+H]+ | 151.0987,55.0542,53.0386 | Dodeca-2(e),4(e)-dienoic acid isobutylamide | C_16_H_29_NO | T3 |
| 170 | 31.26 | 384.2543 | [M+H]+ | 311.1642,283.1698,161.0595,135.0443, 103.0544,77.0388 | Guineensine | C_24_H_33_NO_3_ | T3 |
| 171 | 32.302 | 264.2325 | [M+H]+ | 236.2369,179.1426,138.0914,112.0755, 81.0337,53.0385 | N-cyclohexylundec-9-ynamide | C_17_H_29_NO | T3 |
| 172 | 32.617 | 280.2644 | [M+H]+ | 201.0537,115.0543,55.0540 | Linoleamide | C_18_H_33_NO | T1,T2,T3 |
| 173 | 32.667 | 409.206 | [M-H]- |  | Unidentified |  | T2 |
| 174 | 33.071 | 280.2645 | [M+H]+ |  | Linoleamide | C_18_H_33_NO | T1 |
| 175 | 36.407 | 299.2594 | [M-H]- |  | 9*R*-Hydroxy-octadecanoic acid | C_18_H_36_O_3_ | T1 |

**Table S9** Presents the compounds identified in the Ko-klan remedy formulation-3 (T3), which differ from those found in the formulation-1 (T1) and -2 (T2)

| **No.** | **RT(min)** | **m/z** | **Adduct** | **MS/MS** | **Tentative Identification** | **Formula** |
| --- | --- | --- | --- | --- | --- | --- |
| 44 | 9.294 | 153.0195 | [M-H]- |  | 2,4-Dihydroxybenzoic acid | C_7_H_6_O_4_ |
| 45 | 9.491 | 633.0858 | [M-H]- | 463.0638,301.0011,169.0138 | Punicacortein B | C_27_H_22_O_18_ |
| 52 | 9.994 | 353.0917 | [M-H]- | 271.0546,191.0570,93.0324 | Caffeoyl quinic acid | C_16_H_18_O_9_ |
| 56 | 10.263 | 303.0915 | [M-H]- | 163.0397,123.0455 | 3'-*O*-Methylcatechin | C_16_H_16_O_6_ |
| 57 | 10.488 | 663.1829 | [M-H]- | 627.1979,285.0800,163.0412 | Protosappanin B diglucoside | C_28_H_36_O_16_ |
| 58 | 10.625 | 501.1265 | [M-H]- | 285.0803,163.0401 | Protosappanin B glucoside | C_22_H_26_O_11_ |
| 59 | 10.723 | 322.1288 | [M+NH_4_]+ | 229.0492,165.0698,139.0538,115.0540, 91.0539,68.9966 | Protosappanin B | C_16_H_16_O_6_ |
|  | 10.752 | 303.0907 | [M-H]- | 231.0684,109.0285 | Protosappanin B | C_16_H_16_O_6_ |
| 60 | 10.726 | 287.0916 | [M+H]+ | 271.0582,125.0199 | Brazillin | C_16_H_14_O_5_ |
| 65 | 11.226 | 333.1022 | [M-H]- |  | Methyl 2-(4,4,5'- trihydroxy-2-(methoxymethyl) biphenyl-2-yloxy) acetate | C_17_H_18_O_7_ |
| 66 | 11.303 | 287.092 | [M+H]+ | 269.0798,131.0485,103.0536,77.0382, 51.0224 | (-)-3-Deoxysappanone B | C_16_H_14_O_5_ |
|  | 11.331 | 285.0798 | [M-H]- | 163.0404,121.0292 | (-)-3-Deoxysappanone B | C_16_H_14_O_5_ |
| 68 | 11.363 | 153.0196 | [M-H]- |  | 2,4-Dihydroxybenzoic acid | C_7_H_6_O_4_ |
| 69 | 11.408 | 179.0359 | [M-H]- |  | Caffeic acid | C_9_H_8_O_4_ |
| 72 | 11.515 | 269.0812 | [M+H]+ | 165.0701,115.0541,77.0388,51.0228 | (E)-7-Hydroxy-3-(4-hydroxybenzylidene)chroman-4- one | C_16_H_12_O_4_ |
| 73 | 11.533 | 333.102 | [M-H]- |  | Methyl 2-(4,4,5'- trihydroxy-2-(methoxymethyl) biphenyl-2-yloxy) acetate | C_17_H_18_O_7_ |
| 74 | 11.541 | 303.0931 | [M-H]- | 163.0401,91.0511 | 4'-*O*-Methylcatechin | C_16_H_16_O_6_ |
| 77 | 11.827 | 287.0951 | [M-H]- |  | 3 - Deoxyepisappanol | C_16_H_16_O_5_ |
| 82 | 12.386 | 317.1053 | [M-H]- |  | 3 -*O*-Methylesappanol | C_17_H_18_O_6_ |
| 86 | 12.753 | 301.0029 | [M-H]- | 229.0202,137.0206 | Ellagic acid | C_14_H_6_O_8_ |
| 87 | 12.818 | 285.0795 | [M-H]- | 267.0709,199.0825,109.0290 | Brazillin | C_16_H_14_O_5_ |
| 89 | 13.427 | 285.0759 | [M+H]+ | 257.0796,221.0596,165.0693,147.0438, 102.0460,51.0225 | Sappanone A | C_16_H_12_O_5_ |
|  | 13.453 | 283.0647 | [M-H]- |  | Sappanone A | C1_6_H_12_O_5_ |
| 92 | 13.712 | 315.0549 | [M-H]- |  | Caesalpiniaphenol G | C_16_H_12_O_7_ |
| 94 | 13.838 | 301.0754 | [M-H]- | 179.0354,151.0403,109.0274,65.0389 | Sappanone B | C_16_H_14_O_6_ |
| 96 | 14.284 | 353.0845 | [M+Cl]- |  | 3 -*O*-Methylepisappanol | C_17_H_18_O_6_ |
| 98 | 14.437 | 603.1621 | [2M-H]- | 301.0756,229.0523,159.0450 | Protosappanin C | C_16_H_14_O_6_ |
| 100 | 14.798 | 559.156 | [M-H]- | 397.1190,273.0843,173.0460,93.0359 | Unidentified |  |
| 101 | 15.086 | 603.1623 | [2M-H]- | 301.0763,229.0533,159.0452 | Protosappanin C | C_16_H_14_O_6_ |
| 103 | 15.665 | 287.0916 | [M+H]+ | 257.0428,151.0387,108.0201,89.0382 | Sappanchalcone | C_16_H_14_O_5_ |
|  | 15.68 | 285.08 | [M-H]- | 253.0515,163.0405 | Sappanchalcone | C_16_H_14_O_5_ |
| 106 | 16.181 | 269.081 | [M+H]+ | 176.0613,152.0613,123.0432,103.0536, 77.0380,51.0224 | (*E*)-7-Hydroxy-3-(4-hydroxybenzylidene)chroman-4- one | C_16_H_12_O_4_ |
| 107 | 16.202 | 367.101 | [M+Cl]- | 331.1078,285.0800,163.0408,148.0167,121.0293, 91.0550,65.0384 | Caesalpiniaphenol F | C_18_H_20_O_6_ |
| 108 | 16.203 | 285.0801 | [M-H]- | 163.0401,148.0171,121.0297,93.0346 | (-)-3-Deoxysappanone B | C_16_H_14_O_5_ |
| 109 | 16.232 | 283.0639 | [M-H]- |  | Sappanone A | C_16_H_12_O_5_ |
| 111 | 16.503 | 333.1329 | [M+H]+ | 223.0750,176.0618,152.0615,115.0540, 77.0387,51.0228 | 3 ,4- Di-*O*-methylepisappanol | C_18_H_20_O_6_ |
|  | 16.521 | 367.1011 | [M+Cl]- | 331.1222,163.0406, 121.0646,121.0289 | 3 ,4- Di-*O*-methylepisappanol | C_18_H_20_O_6_ |
| 129 | 21.421 | 272.1288 | [M+H]+ | 201.0541,115.0541, 115.0536,77.0383 | Piperyline | C_16_H_17_NO3 |
| 132 | 22.528 | 276.1592 | [M+H]+ | 201.0551,171.0437,135.0442,77.0387, 51.0228 | Dihydropiperlonguminine | C_16_H_21_NO_3_ |
| 133 | 22.533 | 286.1442 | [M+H]+ |  | Chavicine | C_17_H_19_NO_3_ |
| 134 | 22.663 | 274.144 | [M+H]+ | 201.0549,171.0441,115.0543,77.0389 | Piperlonguminine | C_16_H_19_NO_3_ |
| 135 | 22.656 | 523.3311 | [M+Cl]- | 487.3520,409.1854 | 11-Acetoxy-3β,6α-dihydroxy-24-methylene-9,11-seco-5α-cholesta-7,22E-dien-9-one | C_30_H_48_O_5_ |
| 143 | 24.556 | 349.2216 | [M+Cl]- | 313.2372,201.1158 | 10-Hydroxy-9-ketooctadecanoic acid | C_18_H_34_O_4_ |
| 144 | 24.565 | 302.1754 | [M+H]+ | 167.1307,135.0443,103.0545,77.0388 | (6*E*)-7-(2H-1,3-Benzodioxol-5-YL) -1-(pyrrolidin-1-YL)hept-6-EN-1-one | C_18_H_23_NO_3_ |
| 146 | 25.192 | 271.097 | [M+H]+ | 185.0217,171.0439,147.0440,115.0538, 91.0537,77.0384 | 4,4 -Dihydroxy-2 -methoxychalcone | C_16_H_14_O_4_ |
| 148 | 25.445 | 312.1595 | [M+H]+ | 227.0695,197.0591,164.1071,135.0440, 77.0387 | Piperettine | C_19_H_21_NO_3_ |
| 149 | 25.649 | 314.1751 | [M+H]+ | 227.0700,169.0648,135.042,77.0387 | Piperidine | C_19_H_23_NO_3_ |
| 151 | 26.34 | 293.1892 | [M+Cl]- | 195.1432,96.9616 | 15-Hydroxy-pentadecanoic acid | C_15_H_30_O_3_ |
| 154 | 26.571 | 222.1855 | [M+H]+ | 194.1899,150.0904,124.0754,81.0334 | Sarmentine | C_14_H_23_NO |
| 164 | 28.048 | 571.2816 | [M+H]+ | 486.1921,373.1071,310.1441,192.1020, 86.0965 | Dipiperamide E | C_34_H_38_N_2_O_6_ |
| 165 | 28.397 | 342.2064 | [M+H]+ | 229.1220,135.0442,77.0387 | Pipernonaline | C_21_H_27_NO_3_ |
| 169 | 30.439 | 252.2327 | [M+H]+ | 151.0987,55.0542,53.0386 | Dodeca-2(e),4(e)-dienoic acid isobutylamide | C_16_H_29_NO |
| 170 | 31.26 | 384.2543 | [M+H]+ | 311.1642,283.1698,161.0595,135.0443, 103.0544,77.0388 | Guineensine | C_24_H_33_NO_3_ |
| 171 | 32.302 | 264.2325 | [M+H]+ | 236.2369,179.1426,138.0914,112.0755, | N-Cyclohexylundec-9-ynamide | C_17_H_29_NO |

**Table S10** Molecular docking results between potential compounds and 5-LOX

| **Compound** | **Binding energy (kcal/mol)** |
| --- | --- |
| Sarmentine | −6.26 |
| Pipermonaline | −7.97 |
| Guineensine | −8.27 |
| Piperyline | −7.68 |
| Piperlonguminine | −6.48 |
| Caffeoyl quinic acid | −10.14 |
| Protosappanin B | −9.52 |
| 3-Deoxysappanone B | −9.24 |
| Brazillin | −10.24 |
| 3,4-Di-*O*-methylepisappanol | −9.51 |
| Caesalpiniaphenol F | −9.32 |
| Nordihydroguaiaretic acid (NDGA) | −9.33 |

**Table S11** *P*-values obtained from statistical analyses (one-way ANOVA with Tukey’s post hoc test) comparing iNOS mRNA expressions of Ko-klan remedy extracts

| **Tukey's multiple comparisons test** | **Mean Diff.** | **95% CI of diff.** | **Significant** | **Adjusted P Value** |
| --- | --- | --- | --- | --- |
| Medium vs. LPS | -98.28 | -108.2 to -88.32 | Yes | < 0.0001 |
| Medium vs. Dexa | -37.11 | -47.07 to -27.15 | Yes | < 0.0001 |
| Medium vs. T11 | -62.34 | -72.30 to -52.38 | Yes | < 0.0001 |
| Medium vs. T13 | -38.92 | -48.88 to -28.96 | Yes | < 0.0001 |
| Medium vs. T14 | -58.3 | -68.26 to -48.34 | Yes | < 0.0001 |
| Medium vs. T32 | -87.82 | -97.78 to -77.86 | Yes | < 0.0001 |
| Medium vs. T33 | -72.3 | -82.26 to -62.34 | Yes | < 0.0001 |
| Medium vs. T34 | -83.93 | -93.89 to -73.97 | Yes | < 0.0001 |
| Medium vs. T35 | -47.73 | -57.69 to -37.77 | Yes | < 0.0001 |
| LPS vs. Dexa | 61.17 | 51.21 to 71.13 | Yes | < 0.0001 |
| LPS vs. T11 | 35.94 | 25.98 to 45.90 | Yes | < 0.0001 |
| LPS vs. T13 | 59.36 | 49.40 to 69.32 | Yes | < 0.0001 |
| LPS vs. T14 | 39.98 | 30.02 to 49.94 | Yes | < 0.0001 |
| LPS vs. T32 | 10.46 | 0.4964 to 20.42 | Yes | 0.0348 |
| LPS vs. T33 | 25.98 | 16.02 to 35.94 | Yes | < 0.0001 |
| LPS vs. T34 | 14.35 | 4.386 to 24.31 | Yes | 0.0018 |
| LPS vs. T35 | 50.55 | 40.59 to 60.51 | Yes | < 0.0001 |
| Dexa vs. T11 | -25.23 | -35.19 to -15.27 | Yes | < 0.0001 |
| Dexa vs. T13 | -1.81 | -11.77 to 8.154 | No | 0.9996 |
| Dexa vs. T14 | -21.19 | -31.15 to -11.23 | Yes | < 0.0001 |
| Dexa vs. T32 | -50.71 | -60.67 to -40.75 | Yes | < 0.0001 |
| Dexa vs. T33 | -35.19 | -45.15 to -25.23 | Yes | < 0.0001 |
| Dexa vs. T34 | -46.82 | -56.78 to -36.86 | Yes | < 0.0001 |
| Dexa vs. T35 | -10.62 | -20.58 to -0.6564 | Yes | 0.0309 |
| T11 vs. T13 | 23.42 | 13.46 to 33.38 | Yes | < 0.0001 |
| T11 vs. T14 | 4.04 | -5.924 to 14.00 | No | 0.9013 |
| T11 vs. T32 | -25.48 | -35.44 to -15.52 | Yes | < 0.0001 |
| T11 vs. T33 | -9.96 | -19.92 to 0.00364 | No | 0.0501 |
| T11 vs. T34 | -21.59 | -31.55 to -11.63 | Yes | < 0.0001 |
| T11 vs. T35 | 14.61 | 4.646 to 24.57 | Yes | 0.0014 |
| T13 vs. T14 | -19.38 | -29.34 to -9.416 | Yes | < 0.0001 |
| T13 vs. T32 | -48.9 | -58.86 to -38.94 | Yes | < 0.0001 |
| T13 vs. T33 | -33.38 | -43.34 to -23.42 | Yes | < 0.0001 |
| T13 vs. T34 | -45.01 | -54.97 to -35.05 | Yes | < 0.0001 |
| T13 vs. T35 | -8.81 | -18.77 to 1.154 | No | 0.1115 |
| T14 vs. T32 | -29.52 | -39.48 to -19.56 | Yes | < 0.0001 |
| T14 vs. T33 | -14 | -23.96 to -4.036 | Yes | 0.0023 |
| T14 vs. T34 | -25.63 | -35.59 to -15.67 | Yes | < 0.0001 |
| **Tukey's multiple comparisons test** | **Mean Diff.** | **95% CI of diff.** | **Significant** | **Adjusted P Value** |
| T14 vs. T35 | 10.57 | 0.6064 to 20.53 | Yes | 0.0321 |
| T32 vs. T33 | 15.52 | 5.556 to 25.48 | Yes | 0.0007 |
| T32 vs. T34 | 3.89 | -6.074 to 13.85 | No | 0.919 |
| T32 vs. T35 | 40.09 | 30.13 to 50.05 | Yes | < 0.0001 |
| T33 vs. T34 | -11.63 | -21.59 to -1.666 | Yes | 0.0144 |
| T33 vs. T35 | 24.57 | 14.61 to 34.53 | Yes | < 0.0001 |
| T34 vs. T35 | 36.2 | 26.24 to 46.16 | Yes | < 0.0001 |

**Table S12** *P*-values obtained from statistical analyses (one-way ANOVA with Tukey’s post hoc test) comparing TNF-α mRNA expressions of Ko-klan remedy extracts

| **Tukey's multiple comparisons test** | **Mean Diff.** | **95% CI of diff.** | **Significant?** | **Adjusted P Value** |
| --- | --- | --- | --- | --- |
| Medium vs. LPS | -93.76 | -107.5 to -80.06 | Yes | < 0.0001 |
| Medium vs. Dexa | -47.19 | -60.89 to -33.49 | Yes | < 0.0001 |
| Medium vs. T11 | -90.15 | -103.8 to -76.45 | Yes | < 0.0001 |
| Medium vs. T13 | -83.36 | -97.06 to -69.66 | Yes | < 0.0001 |
| Medium vs. T14 | -85.24 | -98.94 to -71.54 | Yes | < 0.0001 |
| Medium vs. T32 | -121.9 | -135.6 to -108.2 | Yes | < 0.0001 |
| Medium vs. T33 | -96.57 | -110.3 to -82.87 | Yes | < 0.0001 |
| Medium vs. T34 | -78.27 | -91.97 to -64.57 | Yes | < 0.0001 |
| Medium vs. T35 | -52.35 | -66.05 to -38.65 | Yes | < 0.0001 |
| LPS vs. Dexa | 46.57 | 32.87 to 60.27 | Yes | < 0.0001 |
| LPS vs. T11 | 3.61 | -10.09 to 17.31 | No | 0.9931 |
| LPS vs. T13 | 10.4 | -3.299 to 24.10 | No | 0.2426 |
| LPS vs. T14 | 8.52 | -5.179 to 22.22 | No | 0.4858 |
| LPS vs. T32 | -28.14 | -41.84 to -14.44 | Yes | < 0.0001 |
| LPS vs. T33 | -2.81 | -16.51 to 10.89 | No | 0.9989 |
| LPS vs. T34 | 15.49 | 1.791 to 29.19 | Yes | 0.0191 |
| LPS vs. T35 | 41.41 | 27.71 to 55.11 | Yes | < 0.0001 |
| Dexa vs. T11 | -42.96 | -56.66 to -29.26 | Yes | < 0.0001 |
| Dexa vs. T13 | -36.17 | -49.87 to -22.47 | Yes | < 0.0001 |
| Dexa vs. T14 | -38.05 | -51.75 to -24.35 | Yes | < 0.0001 |
| Dexa vs. T32 | -74.71 | -88.41 to -61.01 | Yes | < 0.0001 |
| Dexa vs. T33 | -49.38 | -63.08 to -35.68 | Yes | < 0.0001 |
| Dexa vs. T34 | -31.08 | -44.78 to -17.38 | Yes | < 0.0001 |
| Dexa vs. T35 | -5.16 | -18.86 to 8.539 | No | 0.9334 |
| T11 vs. T13 | 6.79 | -6.909 to 20.49 | No | 0.7535 |
| T11 vs. T14 | 4.91 | -8.789 to 18.61 | No | 0.9497 |
| T11 vs. T32 | -31.75 | -45.45 to -18.05 | Yes | < 0.0001 |
| T11 vs. T33 | -6.42 | -20.12 to 7.279 | No | 0.8043 |
| T11 vs. T34 | 11.88 | -1.819 to 25.58 | No | 0.1247 |
| T11 vs. T35 | 37.8 | 24.10 to 51.50 | Yes | < 0.0001 |
| T13 vs. T14 | -1.88 | -15.58 to 11.82 | No | > 0.9999 |
| T13 vs. T32 | -38.54 | -52.24 to -24.84 | Yes | < 0.0001 |
| T13 vs. T33 | -13.21 | -26.91 to 0.4893 | No | 0.0645 |
| T13 vs. T34 | 5.09 | -8.609 to 18.79 | No | 0.9383 |
| T13 vs. T35 | 31.01 | 17.31 to 44.71 | Yes | < 0.0001 |
| T14 vs. T32 | -36.66 | -50.36 to -22.96 | Yes | < 0.0001 |
| T14 vs. T33 | -11.33 | -25.03 to 2.369 | No | 0.1613 |
| T14 vs. T34 | 6.97 | -6.729 to 20.67 | No | 0.7272 |
| **Tukey's multiple comparisons test** | **Mean Diff.** | **95% CI of diff.** | **Significant?** | **Adjusted P Value** |
| T14 vs. T35 | 32.89 | 19.19 to 46.59 | Yes | < 0.0001 |
| T32 vs. T33 | 25.33 | 11.63 to 39.03 | Yes | < 0.0001 |
| T32 vs. T34 | 43.63 | 29.93 to 57.33 | Yes | < 0.0001 |
| T32 vs. T35 | 69.55 | 55.85 to 83.25 | Yes | < 0.0001 |
| T33 vs. T34 | 18.3 | 4.601 to 32.00 | Yes | 0.0039 |
| T33 vs. T35 | 44.22 | 30.52 to 57.92 | Yes | < 0.0001 |
| T34 vs. T35 | 25.92 | 12.22 to 39.62 | Yes | < 0.0001 |

**Table S13** *P*-values obtained from statistical analyses (one-way ANOVA with Tukey’s post hoc test) comparing COX-2 mRNA expressions of Ko-klan remedy extracts

| **Tukey's multiple comparisons test** | **Mean Diff.** | **95% CI of diff.** | **Significant?** | **Adjusted P Value** |
| --- | --- | --- | --- | --- |
| Medium vs. LPS | -99.85 | -117.1 to -82.56 | Yes | < 0.0001 |
| Medium vs. Dexa | -25.44 | -42.73 to -8.147 | Yes | 0.0014 |
| Medium vs. T11 | -76.1 | -93.39 to -58.81 | Yes | < 0.0001 |
| Medium vs. T13 | -44.98 | -62.27 to -27.69 | Yes | < 0.0001 |
| Medium vs. T14 | -115.1 | -132.4 to -97.80 | Yes | < 0.0001 |
| Medium vs. T32 | -206.2 | -223.5 to -188.9 | Yes | < 0.0001 |
| Medium vs. T33 | -93.78 | -111.1 to -76.49 | Yes | < 0.0001 |
| Medium vs. T34 | -113.6 | -130.9 to -96.31 | Yes | < 0.0001 |
| Medium vs. T35 | -43.67 | -60.96 to -26.38 | Yes | < 0.0001 |
| LPS vs. Dexa | 74.41 | 57.12 to 91.70 | Yes | < 0.0001 |
| LPS vs. T11 | 23.75 | 6.457 to 41.04 | Yes | 0.0029 |
| LPS vs. T13 | 54.87 | 37.58 to 72.16 | Yes | < 0.0001 |
| LPS vs. T14 | -15.24 | -32.53 to 2.053 | No | 0.1137 |
| LPS vs. T32 | -106.3 | -123.6 to -89.02 | Yes | < 0.0001 |
| LPS vs. T33 | 6.07 | -11.22 to 23.36 | No | 0.9555 |
| LPS vs. T34 | -13.75 | -31.04 to 3.543 | No | 0.1963 |
| LPS vs. T35 | 56.18 | 38.89 to 73.47 | Yes | < 0.0001 |
| Dexa vs. T11 | -50.66 | -67.95 to -33.37 | Yes | < 0.0001 |
| Dexa vs. T13 | -19.54 | -36.83 to -2.247 | Yes | 0.0192 |
| Dexa vs. T14 | -89.65 | -106.9 to -72.36 | Yes | < 0.0001 |
| Dexa vs. T32 | -180.7 | -198.0 to -163.4 | Yes | < 0.0001 |
| Dexa vs. T33 | -68.34 | -85.63 to -51.05 | Yes | < 0.0001 |
| Dexa vs. T34 | -88.16 | -105.5 to -70.87 | Yes | < 0.0001 |
| Dexa vs. T35 | -18.23 | -35.52 to -0.9373 | Yes | 0.0337 |
| T11 vs. T13 | 31.12 | 13.83 to 48.41 | Yes | 0.0001 |
| T11 vs. T14 | -38.99 | -56.28 to -21.70 | Yes | < 0.0001 |
| T11 vs. T32 | -130.1 | -147.4 to -112.8 | Yes | < 0.0001 |
| T11 vs. T33 | -17.68 | -34.97 to -0.3873 | Yes | 0.0425 |
| T11 vs. T34 | -37.5 | -54.79 to -20.21 | Yes | < 0.0001 |
| T11 vs. T35 | 32.43 | 15.14 to 49.72 | Yes | < 0.0001 |
| T13 vs. T14 | -70.11 | -87.40 to -52.82 | Yes | < 0.0001 |
| T13 vs. T32 | -161.2 | -178.5 to -143.9 | Yes | < 0.0001 |
| T13 vs. T33 | -48.8 | -66.09 to -31.51 | Yes | < 0.0001 |
| T13 vs. T34 | -68.62 | -85.91 to -51.33 | Yes | < 0.0001 |
| T13 vs. T35 | 1.31 | -15.98 to 18.60 | No | > 0.9999 |
| T14 vs. T32 | -91.07 | -108.4 to -73.78 | Yes | < 0.0001 |
| T14 vs. T33 | 21.31 | 4.017 to 38.60 | Yes | 0.0088 |
| T14 vs. T34 | 1.49 | -15.80 to 18.78 | No | > 0.9999 |
| **Tukey's multiple comparisons test** | **Mean Diff.** | **95% CI of diff.** | **Significant?** | **Adjusted P Value** |
| T14 vs. T35 | 71.42 | 54.13 to 88.71 | Yes | < 0.0001 |
| T32 vs. T33 | 112.4 | 95.09 to 129.7 | Yes | < 0.0001 |
| T32 vs. T34 | 92.56 | 75.27 to 109.9 | Yes | < 0.0001 |
| T32 vs. T35 | 162.5 | 145.2 to 179.8 | Yes | < 0.0001 |
| T33 vs. T34 | -19.82 | -37.11 to -2.527 | Yes | 0.017 |
| T33 vs. T35 | 50.11 | 32.82 to 67.40 | Yes | < 0.0001 |
| T34 vs. T35 | 69.93 | 52.64 to 87.22 | Yes | < 0.0001 |

**Table S14** *P*-values obtained from statistical analyses (one-way ANOVA with Tukey’s post hoc test) comparing IL-1β mRNA expressions of Ko-klan remedy extracts

| **Tukey's multiple comparisons test** | **Mean Diff.** | **95% CI of diff.** | **Significant?** | **Adjusted P Value** |
| --- | --- | --- | --- | --- |
| Medium vs. LPS | -95.82 | -120.5 to -71.12 | Yes | < 0.0001 |
| Medium vs. Dexa | -7.57 | -32.27 to 17.13 | No | 0.9808 |
| Medium vs. T11 | -65.26 | -89.96 to -40.56 | Yes | < 0.0001 |
| Medium vs. T13 | -131 | -155.7 to -106.3 | Yes | < 0.0001 |
| Medium vs. T14 | -92.68 | -117.4 to -67.98 | Yes | < 0.0001 |
| Medium vs. T32 | -93.36 | -118.1 to -68.66 | Yes | < 0.0001 |
| Medium vs. T33 | -60.69 | -85.39 to -35.99 | Yes | < 0.0001 |
| Medium vs. T34 | -100.9 | -125.6 to -76.23 | Yes | < 0.0001 |
| Medium vs. T35 | -45.26 | -69.96 to -20.56 | Yes | < 0.0001 |
| LPS vs. Dexa | 88.25 | 63.55 to 113.0 | Yes | < 0.0001 |
| LPS vs. T11 | 30.56 | 5.855 to 55.26 | Yes | 0.0085 |
| LPS vs. T13 | -35.17 | -59.87 to -10.47 | Yes | 0.002 |
| LPS vs. T14 | 3.14 | -21.56 to 27.84 | No | > 0.9999 |
| LPS vs. T32 | 2.46 | -22.24 to 27.16 | No | > 0.9999 |
| LPS vs. T33 | 35.13 | 10.43 to 59.83 | Yes | 0.002 |
| LPS vs. T34 | -5.11 | -29.81 to 19.59 | No | 0.9989 |
| LPS vs. T35 | 50.56 | 25.86 to 75.26 | Yes | < 0.0001 |
| Dexa vs. T11 | -57.69 | -82.39 to -32.99 | Yes | < 0.0001 |
| Dexa vs. T13 | -123.4 | -148.1 to -98.72 | Yes | < 0.0001 |
| Dexa vs. T14 | -85.11 | -109.8 to -60.41 | Yes | < 0.0001 |
| Dexa vs. T32 | -85.79 | -110.5 to -61.09 | Yes | < 0.0001 |
| Dexa vs. T33 | -53.12 | -77.82 to -28.42 | Yes | < 0.0001 |
| Dexa vs. T34 | -93.36 | -118.1 to -68.66 | Yes | < 0.0001 |
| Dexa vs. T35 | -37.69 | -62.39 to -12.99 | Yes | 0.0009 |
| T11 vs. T13 | -65.73 | -90.43 to -41.03 | Yes | < 0.0001 |
| T11 vs. T14 | -27.42 | -52.12 to -2.715 | Yes | 0.0223 |
| T11 vs. T32 | -28.1 | -52.80 to -3.395 | Yes | 0.0181 |
| T11 vs. T33 | 4.57 | -20.13 to 29.27 | No | 0.9995 |
| T11 vs. T34 | -35.67 | -60.37 to -10.97 | Yes | 0.0017 |
| T11 vs. T35 | 20 | -4.705 to 44.70 | No | 0.1798 |
| T13 vs. T14 | 38.31 | 13.61 to 63.01 | Yes | 0.0007 |
| T13 vs. T32 | 37.63 | 12.93 to 62.33 | Yes | 0.0009 |
| T13 vs. T33 | 70.3 | 45.60 to 95.00 | Yes | < 0.0001 |
| T13 vs. T34 | 30.06 | 5.355 to 54.76 | Yes | 0.0099 |
| T13 vs. T35 | 85.73 | 61.03 to 110.4 | Yes | < 0.0001 |
| T14 vs. T32 | -0.68 | -25.38 to 24.02 | No | > 0.9999 |
| T14 vs. T33 | 31.99 | 7.285 to 56.69 | Yes | 0.0054 |
| T14 vs. T34 | -8.25 | -32.95 to 16.45 | No | 0.967 |
| **Tukey's multiple comparisons test** | **Mean Diff.** | **95% CI of diff.** | **Significant?** | **Adjusted P Value** |
| T14 vs. T35 | 47.42 | 22.72 to 72.12 | Yes | < 0.0001 |
| T32 vs. T33 | 32.67 | 7.965 to 57.37 | Yes | 0.0044 |
| T32 vs. T34 | -7.57 | -32.27 to 17.13 | No | 0.9808 |
| T32 vs. T35 | 48.1 | 23.40 to 72.80 | Yes | < 0.0001 |
| T33 vs. T34 | -40.24 | -64.94 to -15.54 | Yes | 0.0004 |
| T33 vs. T35 | 15.43 | -9.275 to 40.13 | No | 0.4802 |
| T34 vs. T35 | 55.67 | 30.97 to 80.37 | Yes | < 0.0001 |

**Table S15** *P*-values obtained from statistical analyses (one-way ANOVA with Tukey’s post hoc test) comparing IC₅₀ values of nitric oxide (NO) inhibition of Ko-klan remedy extracts

| **Tukey's multiple comparisons test** | **Mean Diff.** | **95% CI of diff.** | **Significant?** | **Adjusted P Value** |
| --- | --- | --- | --- | --- |
| L-NAME vs. T32 | 4.85 | -2.871 to 12.57 | No | 0.2114 |
| L-NAME vs. T33 | 2.01 | -5.711 to 9.731 | No | 0.7174 |
| T32 vs. T33 | -2.84 | -10.56 to 4.881 | No | 0.5329 |

**Table S16** *P*-values obtained from statistical analyses (one-way ANOVA with Tukey’s post hoc test) comparing IC₅₀ values of LOX inhibition of Ko-klan remedy extracts

| **Tukey's multiple comparisons test** | **Mean Diff.** | **95% CI of diff.** | **Significant?** | **Adjusted P Value** |
| --- | --- | --- | --- | --- |
| NDGA vs. T12 | -542.8 | -574.8 to -510.9 | Yes | < 0.0001 |
| NDGA vs. T13 | -584.6 | -616.5 to -552.6 | Yes | < 0.0001 |
| NDGA vs. T14 | -526 | -557.9 to -494.0 | Yes | < 0.0001 |
| NDGA vs. T15 | -366.6 | -398.5 to -334.6 | Yes | < 0.0001 |
| NDGA vs. T21 | -501.4 | -533.3 to -469.4 | Yes | < 0.0001 |
| NDGA vs. T22 | -463.7 | -495.6 to -431.7 | Yes | < 0.0001 |
| NDGA vs. T23 | -371.4 | -403.4 to -339.5 | Yes | < 0.0001 |
| NDGA vs. T24 | -427 | -458.9 to -395.0 | Yes | < 0.0001 |
| NDGA vs. T32 | -77.33 | -109.3 to -45.37 | Yes | < 0.0001 |
| NDGA vs. T33 | -92.18 | -124.1 to -60.22 | Yes | < 0.0001 |
| NDGA vs. T34 | -60.96 | -92.92 to -29.00 | Yes | < 0.0001 |
| NDGA vs. T35 | -138.5 | -170.4 to -106.5 | Yes | < 0.0001 |
| T12 vs. T13 | -41.73 | -73.69 to -9.773 | Yes | 0.0036 |
| T12 vs. T14 | 16.87 | -15.09 to 48.83 | No | 0.7711 |
| T12 vs. T15 | 176.3 | 144.3 to 208.2 | Yes | < 0.0001 |
| T12 vs. T21 | 41.47 | 9.513 to 73.43 | Yes | 0.0038 |
| T12 vs. T22 | 79.13 | 47.17 to 111.1 | Yes | < 0.0001 |
| T12 vs. T23 | 171.4 | 139.4 to 203.4 | Yes | < 0.0001 |
| T12 vs. T24 | 115.8 | 83.87 to 147.8 | Yes | < 0.0001 |
| T12 vs. T32 | 465.5 | 433.5 to 497.4 | Yes | < 0.0001 |
| T12 vs. T33 | 450.6 | 418.7 to 482.6 | Yes | < 0.0001 |
| T12 vs. T34 | 481.9 | 449.9 to 513.8 | Yes | < 0.0001 |
| T12 vs. T35 | 404.4 | 372.4 to 436.3 | Yes | < 0.0001 |
| T13 vs. T14 | 58.6 | 26.64 to 90.56 | Yes | < 0.0001 |
| T13 vs. T15 | 218 | 186.0 to 250.0 | Yes | < 0.0001 |
| T13 vs. T21 | 83.2 | 51.24 to 115.2 | Yes | < 0.0001 |
| T13 vs. T22 | 120.9 | 88.90 to 152.8 | Yes | < 0.0001 |
| T13 vs. T23 | 213.1 | 181.2 to 245.1 | Yes | < 0.0001 |
| T13 vs. T24 | 157.6 | 125.6 to 189.5 | Yes | < 0.0001 |
| T13 vs. T32 | 507.2 | 475.3 to 539.2 | Yes | < 0.0001 |
| T13 vs. T33 | 492.4 | 460.4 to 524.3 | Yes | < 0.0001 |
| T13 vs. T34 | 523.6 | 491.6 to 555.5 | Yes | < 0.0001 |
| T13 vs. T35 | 446.1 | 414.1 to 478.1 | Yes | < 0.0001 |
| T14 vs. T15 | 159.4 | 127.4 to 191.4 | Yes | < 0.0001 |
| T14 vs. T21 | 24.6 | -7.357 to 56.56 | No | 0.2613 |
| T14 vs. T22 | 62.26 | 30.30 to 94.22 | Yes | < 0.0001 |
| T14 vs. T23 | 154.5 | 122.6 to 186.5 | Yes | < 0.0001 |
| T14 vs. T24 | 98.96 | 67.00 to 130.9 | Yes | < 0.0001 |
| **Tukey's multiple comparisons test** | **Mean Diff.** | **95% CI of diff.** | **Significant?** | **Adjusted P Value** |
| T14 vs. T32 | 448.6 | 416.7 to 480.6 | Yes | < 0.0001 |
| T14 vs. T33 | 433.8 | 401.8 to 465.7 | Yes | < 0.0001 |
| T14 vs. T34 | 465 | 433.0 to 496.9 | Yes | < 0.0001 |
| T14 vs. T35 | 387.5 | 355.5 to 419.5 | Yes | < 0.0001 |
| T15 vs. T21 | -134.8 | -166.8 to -102.8 | Yes | < 0.0001 |
| T15 vs. T22 | -97.14 | -129.1 to -65.18 | Yes | < 0.0001 |
| T15 vs. T23 | -4.87 | -36.83 to 27.09 | No | > 0.9999 |
| T15 vs. T24 | -60.44 | -92.40 to -28.48 | Yes | < 0.0001 |
| T15 vs. T32 | 289.2 | 257.3 to 321.2 | Yes | < 0.0001 |
| T15 vs. T33 | 274.4 | 242.4 to 306.3 | Yes | < 0.0001 |
| T15 vs. T34 | 305.6 | 273.6 to 337.5 | Yes | < 0.0001 |
| T15 vs. T35 | 228.1 | 196.1 to 260.1 | Yes | < 0.0001 |
| T21 vs. T22 | 37.66 | 5.703 to 69.62 | Yes | 0.0111 |
| T21 vs. T23 | 129.9 | 97.97 to 161.9 | Yes | < 0.0001 |
| T21 vs. T24 | 74.36 | 42.40 to 106.3 | Yes | < 0.0001 |
| T21 vs. T32 | 424 | 392.1 to 456.0 | Yes | < 0.0001 |
| T21 vs. T33 | 409.2 | 377.2 to 441.1 | Yes | < 0.0001 |
| T21 vs. T34 | 440.4 | 408.4 to 472.3 | Yes | < 0.0001 |
| T21 vs. T35 | 362.9 | 330.9 to 394.9 | Yes | < 0.0001 |
| T22 vs. T23 | 92.27 | 60.31 to 124.2 | Yes | < 0.0001 |
| T22 vs. T24 | 36.7 | 4.743 to 68.66 | Yes | 0.0144 |
| T22 vs. T32 | 386.4 | 354.4 to 418.3 | Yes | < 0.0001 |
| T22 vs. T33 | 371.5 | 339.6 to 403.5 | Yes | < 0.0001 |
| T22 vs. T34 | 402.7 | 370.8 to 434.7 | Yes | < 0.0001 |
| T22 vs. T35 | 325.2 | 293.3 to 357.2 | Yes | < 0.0001 |
| T23 vs. T24 | -55.57 | -87.53 to -23.61 | Yes | < 0.0001 |
| T23 vs. T32 | 294.1 | 262.1 to 326.0 | Yes | < 0.0001 |
| T23 vs. T33 | 279.2 | 247.3 to 311.2 | Yes | < 0.0001 |
| T23 vs. T34 | 310.5 | 278.5 to 342.4 | Yes | < 0.0001 |
| T23 vs. T35 | 233 | 201.0 to 264.9 | Yes | < 0.0001 |
| T24 vs. T32 | 349.7 | 317.7 to 381.6 | Yes | < 0.0001 |
| T24 vs. T33 | 334.8 | 302.9 to 366.8 | Yes | < 0.0001 |
| T24 vs. T34 | 366 | 334.1 to 398.0 | Yes | < 0.0001 |
| T24 vs. T35 | 288.5 | 256.6 to 320.5 | Yes | < 0.0001 |
| T32 vs. T33 | -14.85 | -46.81 to 17.11 | No | 0.882 |
| T32 vs. T34 | 16.37 | -15.59 to 48.33 | No | 0.8018 |
| T32 vs. T35 | -61.12 | -93.08 to -29.16 | Yes | < 0.0001 |
| T33 vs. T34 | 31.22 | -0.7369 to 63.18 | No | 0.0601 |
| T33 vs. T35 | -46.27 | -78.23 to -14.31 | Yes | 0.001 |
| T34 vs. T35 | -77.49 | -109.4 to -45.53 | Yes | < 0.0001 |
